# Left and right myocardial performance indices in growth‐restricted fetuses: systematic review and meta‐analysis

**DOI:** 10.1002/uog.70233

**Published:** 2026-05-11

**Authors:** A. Sirico, I. Mappa, G. M. Maruotti, L. Cobellis, G. Rizzo

**Affiliations:** ^1^ Department of Woman, Child, and General and Specialized Surgery University of Campania ‘Luigi Vanvitelli’ Naples Italy; ^2^ Department of Maternal and Child Health and Urological Sciences Sapienza University of Rome Rome Italy; ^3^ Obstetrics and Gynecology Unit, Department of Public Health University of Naples Federico II Naples Italy

**Keywords:** diastole, echocardiography, Doppler, fetal growth restriction, perinatal outcome, placental insufficiency, Tei index, ventricular dysfunction, left, ventricular dysfunction, right

## Abstract

**Objectives:**

To evaluate alterations in fetal cardiac function in pregnancies complicated by fetal growth restriction (FGR) by synthesizing evidence on myocardial performance index (MPI) and its constituent parameters for both the left and right ventricles. The secondary objective was to explore the influence of timing of FGR onset (early *vs* late) on these parameters.

**Methods:**

We conducted a systematic review and meta‐analysis of observational studies by searching PubMed/MEDLINE, EMBASE, Scopus, Web of Science and the Cochrane Central Register of Controlled Trials (CENTRAL) databases from inception until August 2025. Eligible studies compared fetal MPI, isovolumetric contraction time (ICT), ejection time (ET), isovolumetric relaxation time (IRT) and/or peak early‐to‐late diastolic filling velocity ratio (E/A ratio) between FGR fetuses and healthy controls. Data reported in original publications as median with interquartile range, range or 95% CI were converted to mean ± SD. A random‐effects model was used to calculate the pooled standardized mean difference (SMD) with 95% CI. Prespecified subgroup analyses were performed based on ventricular laterality, timing of FGR onset and data presentation type. The protocol was registered with PROSPERO (registration number: CRD420251075193).

**Results:**

Fifteen studies, comprising 709 FGR fetuses and 867 controls, were included. Compared with controls, FGR fetuses exhibited significant left ventricular dysfunction, characterized by a higher left MPI (SMD, 0.85 (95% CI, 0.53–1.16)), prolonged left ICT (SMD, 0.53 (95% CI, 0.08–0.99)) and shorter left ET (SMD, −0.56 (95% CI, −0.84 to −0.29)). The most profound alteration was a prolongation of the left IRT in FGR cases (SMD, 2.48 (95% CI, 1.55–3.41)). Right ventricular assessment revealed a prolonged right IRT in FGR fetuses compared with controls (SMD, 1.90 (95% CI, 0.72–3.07)). Subgroup analysis showed that myocardial functional alterations affect both early‐ and late‐onset FGR phenotypes, with early cases showing a non‐significant trend toward more pronounced impairment. Sensitivity analysis including only studies that employed stricter diagnostic criteria for FGR confirmed these findings. Significant publication bias was detected for the analysis of left MPI and left IRT.

**Conclusions:**

FGR is associated with significant biventricular cardiac dysfunction, characterized primarily by impaired myocardial relaxation, as indicated by a markedly prolonged IRT. The IRT appears to be a more sensitive marker of cardiac compromise in FGR compared with the traditional E/A ratio. These findings support the use of MPI and its components as valuable adjunctive tools in the surveillance of FGR pregnancies. © 2026 The Author(s). *Ultrasound in Obstetrics & Gynecology* published by John Wiley & Sons Ltd on behalf of International Society of Ultrasound in Obstetrics and Gynecology.

## INTRODUCTION

Fetal growth restriction (FGR), a condition affecting approximately 5–7% of pregnancies globally, represents one of the most significant challenges in modern obstetrics^1^, ^2^. FGR is associated with a substantial increase in the risk of adverse perinatal outcomes, including perinatal mortality and a wide spectrum of neonatal morbidities, such as birth asphyxia, respiratory distress syndrome, necrotizing enterocolitis and intraventricular hemorrhage^3^, ^4^. Despite advances in prenatal surveillance, the perinatal morbidity rate associated with FGR remains alarmingly high, underscoring a persistent gap in our ability to promptly identify fetuses at greatest risk and to optimize the timing of delivery^5^, ^6^.

The pathophysiology of FGR is complex, but, in most instances, placental insufficiency is recognized as the primary underlying mechanism. This dysfunction leads to a state of chronic hypoxia and metabolic stress^7^. In response to these conditions, adaptive mechanisms are activated in the fetus, including the preferential redistribution of blood flow towards vital organs, such as the brain, heart and adrenal glands^8^. This hemodynamic compensation, while initially protective, can itself contribute to a progressive deterioration of fetal cardiac function and myocardial remodeling, potentially laying the groundwork for cardiovascular disease in adulthood, in line with Barker's hypothesis on the fetal programming of adult disease^9^, ^10^.

In this context, advanced fetal echocardiography has assumed an increasingly central role in assessing fetal wellbeing. The myocardial performance index (MPI), also known as the Tei index, has emerged as a promising tool for the non‐invasive evaluation of global fetal cardiac function, integrating both systolic and diastolic performance^11^. The MPI is defined as the sum of the isovolumetric contraction time (ICT) and isovolumetric relaxation time (IRT), divided by the ejection time (ET)^12^. An increase in MPI typically reflects a deterioration in cardiac function, often due to a prolongation of the isovolumetric periods and/or a reduction in ET. One of the main advantages of the MPI lies in its relative independence from ventricular geometry and fetal heart rate, as well as its theoretical ease of acquisition during a standard Doppler ultrasound examination^13^, ^14^. To overcome some of the reproducibility‐related limitations of the original technique, a modified version of the MPI (Mod‐MPI) was introduced, which uses the opening and closing ‘clicks’ of the heart valves as more precise temporal landmarks^15^.

In recent years, numerous studies have investigated the role of MPI in the assessment of FGR fetuses, and have suggested that this index may be altered early in gestation in response to chronic hypoxia and hemodynamic stress. However, the existing literature is characterized by considerable heterogeneity in FGR definitions, inclusion criteria and reported results.

The objective of this systematic review and meta‐analysis was to evaluate the differences in MPI, its constituent parameters (ICT, ET, IRT) and the peak early‐to‐late diastolic filling velocity ratio (E/A ratio), for both the left and right ventricles, between fetuses diagnosed with FGR and controls. The secondary objective was to explore the influence of the timing of FGR onset (early *vs* late) on these parameters.

## METHODS

This systematic review and meta‐analysis was designed prospectively and was conducted and reported in accordance with the Preferred Reporting Items for Systematic Reviews and Meta‐Analyses (PRISMA) guidelines^16^. The protocol for this review was registered with the International Prospective Register of Systematic Reviews (PROSPERO) (registration number: CRD420251075193).

### Eligibility criteria

We included observational studies, specifically those with a cohort, case–control or cross‐sectional design, published as full‐text articles in the English language. Cases were pregnant women with a fetus diagnosed with FGR or intrauterine growth restriction, and controls were pregnant women with a healthy, appropriately grown fetus. Included studies defined FGR based on established criteria, such as an estimated fetal weight (EFW) < 10^th^ percentile, often corroborated by abnormal Doppler velocimetry or other consensus‐based definitions. Included studies performed fetal echocardiographic assessment that reported on the MPI, its individual components (ICT, IRT or ET) and/or the E/A ratio. The primary outcomes sought were the mean ± SD values for these parameters for both the left and right ventricles.

Studies were excluded if they were animal studies, review articles, editorials, Letters to the Editor or case reports. Furthermore, studies lacking a control group and those that did not report data in a format amenable to meta‐analysis (i.e. not providing mean ± SD or sufficient data for their estimation) were excluded. We also excluded studies that compared MPI between subtypes of FGR rather than between FGR and controls.

### Information sources and search strategy

A systematic and comprehensive literature search was performed across several electronic databases, including PubMed/MEDLINE, EMBASE, Scopus, Web of Science and the Cochrane Central Register of Controlled Trials (CENTRAL). The search was conducted from the inception of each database until August 2025 without language restrictions initially, although only full‐text articles in the English language were ultimately included. The search strategy employed a combination of medical subject headings (MeSH) (or equivalent) and keywords related to ‘fetal growth restriction’, ‘intrauterine growth restriction’, ‘small‐for‐gestational age’, ‘myocardial performance index’, ‘Tei index’, ‘fetal echocardiography’ and ‘cardiac function’, as well as the individual components of MPI (ICT, IRT, ET) and the E/A ratio. To ensure comprehensiveness, the reference lists of all retrieved articles and relevant review articles were screened manually for additional potentially eligible studies.

### Study selection and data extraction

Study selection was performed by two independent reviewers (A.S., I.M.). Initially, the title and abstract of all records identified by the literature search were screened against the predefined eligibility criteria. Subsequently, the full‐text version of all potentially relevant studies was retrieved and subjected to detailed assessment by the same two independent reviewers. Discrepancy or uncertainty regarding study inclusion was resolved through discussion between the two reviewers or, if consensus could not be reached, by consultation with a third reviewer (G.R.).

Following study selection, data were extracted independently by two reviewers (A.S., I.M.) using a pre‐piloted, standardized data extraction form. This form captured key information including: first author, year of publication, study location, study design, number of cases and controls, gestational‐age range at the time of MPI assessment, diagnostic criteria for FGR and primary outcome data for cases and controls. Disagreements during data extraction were resolved by discussion between the two reviewers or by consultation with a third reviewer (G.R.). For studies with missing or ambiguously reported data, we attempted to contact the corresponding author by e‐mail to request the raw mean ± SD values.

For studies that presented continuous data as median with interquartile range (IQR), range or 95% CI, a data conversion was performed to estimate the mean and SD. This crucial step was guided primarily by the validated methods described by Wan *et al*.^17^ and Luo *et al*.^18^. Specifically, when the median, first quartile (Q1), third quartile (Q3) and sample size (*n*) were available, the mean was estimated using the formula: mean ≈ (Q1 + (2 × median) + Q3) / 4. The corresponding SD was estimated using the formula: SD ≈ (Q3 − Q1) / (2 × Φ^−1^((0.75*n* − 0.125) / (*n* + 0.25))), where Φ^−1^ represents the inverse of the standard normal cumulative distribution function.

### Risk‐of‐bias assessment

The methodological quality and potential for bias in each included study were assessed independently by two reviewers (A.S., I.M.). This assessment used a domain‐based approach, with criteria adapted from established frameworks for evaluating non‐randomized (observational) studies, such as the Cochrane Risk of Bias in Non‐randomized Studies of Interventions (ROBINS‐I) tool, tailored to the specific context of studies comparing fetal cardiac parameters^19^, ^20^. For each study, the following five key domains were evaluated.

#### Bias due to confounding

This domain assessed the extent to which the relationship between FGR and cardiac parameters might be distorted by other factors associated with both FGR and cardiac function. Reviewers considered whether studies identified and accounted appropriately for key potential confounders in their design and analysis. Important confounders included, but were not limited to, gestational age at assessment, maternal age, maternal body mass index, pre‐existing maternal medical conditions (e.g. hypertension or diabetes unrelated to the primary FGR exposure group, to assess whether controls were truly ‘healthy’) and smoking status. Classification as low risk of bias required clear identification and adequate control (e.g. matching, stratification or multivariable adjustment) for major confounders.

#### Bias in selection of participants

This domain focused on the methods used to select cases and controls, and whether these methods could have introduced systematic differences between the groups beyond FGR status. For cases, criteria included the clarity and consistency of the FGR definition and the representativeness of the recruited sample. For controls, criteria included selection from the same source population as cases, suitability for comparison (e.g. confirmed appropriate fetal growth) and methods to minimize selection bias (e.g. consecutive or random recruitment, if applicable).

#### Bias in measurement of exposure and outcome

This domain evaluated the reliability and validity of how FGR status (exposure) was determined and how cardiac parameters (outcomes) were measured. This included assessing whether objective and standardized criteria were used for FGR diagnosis, whether MPI measurements were performed by trained personnel using standardized protocols, whether assessors were blinded to FGR status (where feasible), and whether appropriate methods were used to ensure the consistency of measurements (e.g. averaging multiple readings, reporting intra‐/interobserver variability).

#### Bias due to missing data (attrition bias)

This domain considered the extent of missing data for outcomes or important baseline variables, the reasons for missing data (if reported) and how missing data were handled in the analysis. A low risk of bias was assigned if missing data were minimal or if appropriate statistical methods (e.g. multiple imputation) were used to address missing data, with evidence that missingness was unlikely to be related to the true value of the missing outcome or variable.

#### Bias in reporting of results (selective reporting bias)

This domain assessed the likelihood that studies selectively reported only some of their findings based on statistical significance or direction of effect. Reviewers looked for evidence of a prespecified analysis plan (e.g. study protocol), reporting of all relevant measured cardiac parameters (including those that were non‐significant), and consistency between the methods and results.

For each domain, a judgment of low, unclear or high risk of bias was assigned based on predefined signaling questions and criteria. Disagreement between the two reviewers was resolved through discussion until consensus was achieved. The overall risk of bias for each study was not scored quantitatively; instead, the pattern of risk scores across domains was considered qualitatively when interpreting the meta‐analysis results and planning the sensitivity analysis.

### Data trustworthiness assessment

To ensure the integrity of the synthesized evidence, *post‐hoc* trustworthiness screening was conducted for all eligible studies, using criteria adapted from the Cochrane Pregnancy and Childbirth Trustworthiness Screening Tool^21^. We systematically screened the publication history of the corresponding author and first author of all included studies for retractions or expressions of concern by querying both the Retraction Watch database (https://retractiondatabase.org/) and PubMed/MEDLINE. Additionally, for studies originating from the same country or institution, we cross‐referenced recruitment periods and hospital affiliations to exclude overlapping datasets. Studies that were flagged for data integrity concerns or authored by individuals with a record of retraction(s) due to data unreliability were excluded.

### Statistical analysis

Meta‐analysis was performed using Review Manager (RevMan) software (version 5.4; The Cochrane Collaboration, Copenhagen, Denmark). The primary summary statistic was the standardized mean difference (SMD) with its corresponding 95% CI. SMD was chosen as the effect measure to account for potential variation in measurement scales and the inherent variability of cardiac parameters across different studies, even when assessing the same nominal cardiac index. Due to anticipated clinical and methodological heterogeneity among the included studies, a random‐effects model, as proposed by DerSimonian and Laird, was used for all pooled analyses^22^. For meta‐analysis results, a *P*‐value < 0.05 was set as the threshold for statistical significance.

Statistical heterogeneity across studies was quantified using Cochran's Q test, with a *P*‐value < 0.10 considered indicative of significant heterogeneity. The percentage of the total variation in pooled estimates that was due to heterogeneity rather than chance was further assessed using the *I* ^2^ statistic, which was interpreted as follows: < 25%, low heterogeneity; 25–50%, moderate heterogeneity; and > 50%, high heterogeneity^23^.

To explore potential sources of heterogeneity and provide more nuanced insights, prespecified subgroup analyses were conducted for each cardiac parameter. These analyses were stratified by: (1) ventricular laterality, comparing cardiac parameters specific to the left ventricle and those specific to the right ventricle; (2) data presentation type, comparing studies that originally reported outcomes as mean ± SD with those for which data were converted from other formats; and (3) gestational age at FGR diagnosis, comparing studies focusing primarily on early‐onset FGR (diagnosis or enrollment predominantly before 32–34 weeks' gestation) with those focusing on late‐onset FGR (diagnosis or enrollment predominantly at or after 32–34 weeks). The gestational age cut‐off for the third subgroup analysis was determined based on the population characteristics reported by each study.

In addition, a sensitivity analysis was conducted to assess the robustness of our findings in relation to FGR diagnostic criteria. This analysis included only the subset of studies that employed the stricter FGR definition proposed by a Delphi consensus^1^ (abdominal circumference (AC)/EFW < 3^rd^ percentile, or AC/EFW < 10^th^ percentile in conjunction with abnormal Doppler velocimetry or slow fetal growth), either in its entirety or in part. Therefore, studies that defined FGR solely as AC/EFW < 10^th^ percentile were excluded from this analysis.

Publication bias was investigated for each primary outcome measure by visual inspection of funnel plots, provided that at least 10 studies were included in the respective meta‐analysis. When sufficient studies were available, Egger's linear regression test was used as a more formal statistical assessment of funnel plot asymmetry^24^.

## RESULTS

### Study selection and characteristics

**Figure 1:**
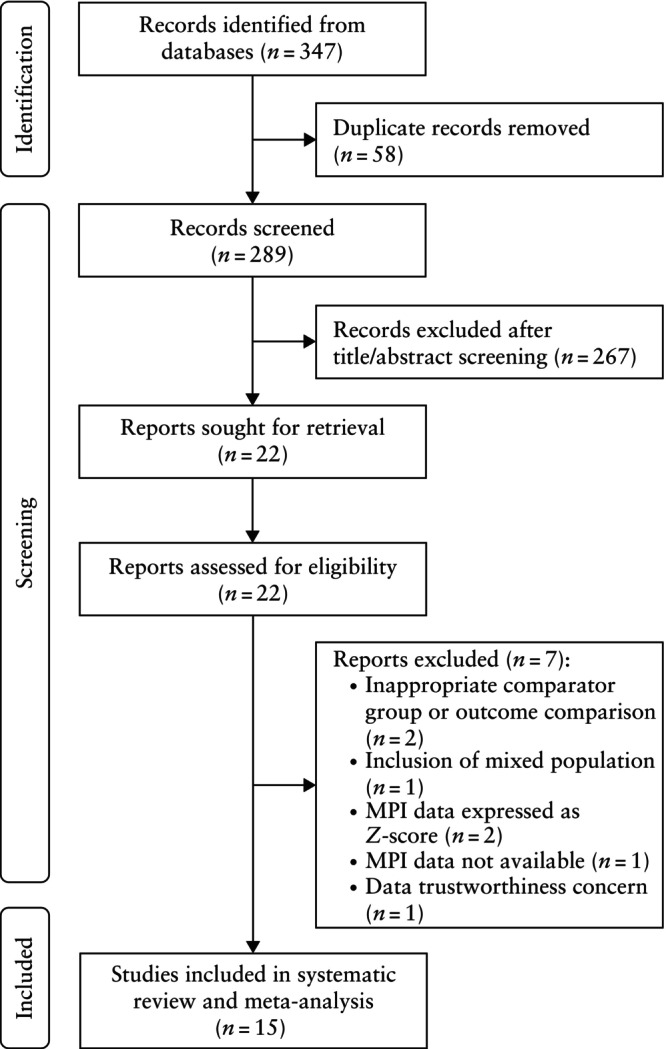
Flowchart summarizing identification, screening, eligibility assessment and inclusion of studies in systematic review and meta‐analysis. MPI, myocardial performance index.

The initial systematic literature search yielded 347 records (Figure 1). After the removal of 58 duplicate records, 289 unique records underwent title and abstract screening, and 22 full‐text articles were retrieved for detailed eligibility assessment^25^, ^26^, ^27^, ^28^, ^29^, ^30^, ^31^, ^32^, ^33^, ^34^, ^35^, ^36^, ^37^, ^38^, ^39^, ^40^, ^41^, ^42^, ^43^, ^44^, ^45^, ^46^. Upon full‐text review, seven articles were excluded^25^, ^26^, ^27^, ^28^, ^29^, ^30^, ^31^. The primary reasons for exclusion at this stage were the use of an inappropriate comparator group or an outcome comparison methodology that did not align with the objectives of this meta‐analysis^26^, ^28^; inclusion of a mixed population of small‐for‐gestational‐age and FGR fetuses without disaggregated data^30^; and reporting of MPI data mostly as *Z*‐scores, which precluded direct extraction of mean ± SD values, as raw data could not be obtained from the original authors upon request^27^, ^29^. Additionally, the study of Chawengsettakul *et al*.^25^ did not provide retrievable MPI data for the FGR group. Finally, after the systematic trustworthiness assessment, we excluded the study of Nassr *et al*.^31^ because of the first author's record of retractions in other journals, which raised concerns about data reliability (Table S1).

We identified eight studies from the same country (Turkey)^36^, ^38^, ^40^, ^41^, ^42^, ^43^, ^45^, ^46^. A detailed evaluation of hospital affiliations and recruitment windows confirmed that these investigations involved distinct patient populations with non‐overlapping recruitment periods. The reported sample sizes were deemed plausible relative to the study settings; therefore, these datasets were considered trustworthy and were retained in our analysis.

Consequently, 15 primary studies fulfilled all predefined eligibility criteria and were included in our systematic review and meta‐analysis^32^, ^33^, ^34^, ^35^, ^36^, ^37^, ^38^, ^39^, ^40^, ^41^, ^42^, ^43^, ^44^, ^45^, ^46^. The characteristics of the included studies are summarized in Table 1 and detailed in Table S2.

**Table 1:** Characteristics of 15 studies included in systematic review and meta‐analysis of myocardial performance index in fetal growth restriction (FGR)

Study	Location	Study design	Cases (*n*)	Controls (*n*)	GA at US (weeks)	Exclusion criteria	FGR diagnostic criteria	EFW chart
*LV parameters*								
Comas (2010)^32^	Spain	Prosp case–control	25	50	26–34	Fetal anomaly*, infection	EFW < p10 + UA‐PI > p95	Figueras^53^
Hassan (2013)^33^	UK	Prosp observational	12	48	24–32	Fetal anomaly, aneuploidy, infection	AC < p5 + abnormal UA Doppler	Hadlock^54^
Pacheco Silva (2016)^34^	Brazil	Prosp case–control	22	24	24–34	Fetal anomaly*	EFW < p3	Hadlock^54^
Henry (2018)^35^	Australia	Cohort/nested case–control	52	52	24–38	Fetal anomaly*, multiple pregnancy, BW ≥ p10	EFW < p10, or AC < p10 + abnormal UA Doppler	N/A
Öcal (2019)^36^	Turkey	Prosp case–control	40	40	29–39	Maternal systemic disease, fetal anomaly*	EFW < p10	Hadlock^54^
Patey (2019)^37^	UK	Prosp cohort	33	54	≥ 37	Multiple pregnancy, fetal anomaly*, maternal disease, women in labor	EFW < p10 + Doppler signs of placental dysfunction	N/A
Zhang (2019)^39^	China	Prosp case–control	21	100	< 32	Multiple pregnancy, fetal anomaly, maternal disease	EFW < p10 + abnormal fetal Doppler	Hadlock†
Zhang (2019)^39^	China	Prosp case–control	56	100	< 32	Multiple pregnancy, fetal anomaly, maternal disease	EFW < p10 + normal fetal Doppler	Hadlock†
Zhang (2019)^39^	China	Prosp case–control	13	100	≥ 32	Multiple pregnancy, fetal anomaly, maternal disease	EFW < p10 + abnormal fetal Doppler	Hadlock†
Zhang (2019)^39^	China	Prosp case–control	87	100	≥ 32	Multiple pregnancy, fetal anomaly, maternal disease	EFW < p10 + normal fetal Doppler	Hadlock†
Davutoglu (2020)^40^	Turkey	Prosp case–control	22	34	< 34	PE, DM, maternal chronic disease, fetal anomaly, multiple pregnancy	EFW < p10	Hadlock^55^
Davutoglu (2020)^40^	Turkey	Prosp case–control	51	32	≥ 34	PE, DM, maternal chronic disease, fetal anomaly, multiple pregnancy	EFW < p10	Hadlock^55^
Jain (2022)^44^	India	Prosp cohort	44	48	N/A	Multiple pregnancy, fetal anomaly*, abnormal fetal heart rate	AC/EFW < p3 or AC/EFW p3–p10 + abnormal Doppler	N/A
Turkyilmaz (2022)^42^	Turkey	Prosp case–control	28	28	32–37	Hypertensive pregnancy, GDM, fetal anomaly	AC/EFW < p3 or at least two of AC/EFW < p10, AC/EFW crossing percentiles by > 2 SD, CPR < p5 or UA‐PI > p95	Hadlock†
Yakut (2022)^43^	Turkey	Prosp case–control	9	54	< 32	Maternal systemic disease, fetal anomaly*, multiple pregnancy, drug use, PPROM, chorioamnionitis	EFW < p3 or EFW < p10 + UA‐PI > p95 or UA‐AEDF/UA‐REDF/CPR < 1	N/A
Yakut (2022)^43^	Turkey	Prosp case–control	21	54	≥ 32	Maternal systemic disease, fetal anomaly*, multiple pregnancy, drug use, PPROM, chorioamnionitis	EFW < p3 or EFW < p10 + UA‐PI > p95 or UA‐AEDF/UA‐REDF/CPR < 1	N/A
Oluklu (2023)^45^	Turkey	Prosp case–control	28	28	< 32	Maternal systemic disease, drug use, nutritional disorder, smoking, umbilical cord or placental anomaly, fetal anomaly*	EFW < p10	Hadlock^55^
Oluklu (2023)^45^	Turkey	Prosp case–control	54	54	≥ 32	Maternal systemic disease, drug use, nutritional disorder, smoking, umbilical cord or placental anomaly, fetal anomaly*	EFW < p10	Hadlock^55^
Dal (2024)^46^	Turkey	Retro	21	35	≥ 32	Multiple pregnancy, structural/placental anomaly, GDM, polyhydramnios, chorioamnionitis, PPROM, chronic systemic disease, drug use, aneuploidy, syndromes	AC/EFW < p10	FMF^56^
*RV parameters*								
Henry (2018)^35^	Australia	Cohort/nested case–control	52	52	24–38	Fetal anomaly*, multiple pregnancy, BW ≥ p10	EFW < p10, or AC < p10 + abnormal UA Doppler	N/A
Kaya (2019)^38^	Turkey	Prosp case–control	40	40	34–37	Maternal systemic disease, fetal anomaly*, multiple pregnancy, arrhythmia, fetal infection, tobacco use	EFW < p3 or EFW < p10 + abnormal Doppler (UA/CPR/UtA‐PI)	N/A
Patey (2019)^37^	UK	Prosp cohort	33	54	≥ 37	Multiple pregnancy, fetal anomaly*, maternal disease, women in labor	EFW < p10 + Doppler signs of placental dysfunction	N/A
Palalioglu (2021)^41^	Turkey	Prosp case–control	30	46	24–34	Multiple pregnancy, fetal anomaly*, maternal disease, oligohydramnios, PROM	EFW < p3 or EFW < p10 + abnormal Doppler (UA/CPR/UtA‐PI)	N/A

Only first author is given for each study.

* Structural or chromosomal anomaly.

† Reference not provided by original study. AC, abdominal circumference; AEDF, absent end‐diastolic flow; BW, birth weight; CPR, cerebroplacental ratio; DM, diabetes mellitus; EFW, estimated fetal weight; FMF, Fetal Medicine Foundation; GA, gestational age; GDM, gestational diabetes mellitus; LV, left ventricular; N/A, not available or not reported; p3/p5/p10/p95, 3^rd^/5^th^/10^th^/95^th^ percentile; PE, pre‐eclampsia; PI, pulsatility index; PPROM, preterm prelabor rupture of membranes; PROM, prelabor rupture of membranes; Prosp, prospective; REDF, reversed end‐diastolic flow; Retro, retrospective; RV, right ventricular; UA, umbilical artery; US, ultrasound; UtA, uterine artery.

Published between 2010 and 2024, the 15 selected studies collectively contributed data from a diverse international FGR cohort. The total number of unique participants across all included studies was 709 FGR fetuses and 867 controls. Several studies reported data for distinct subgroups based on gestational age at FGR diagnosis (early *vs* late onset) and/or the presence of Doppler abnormalities^39^, ^40^, ^43^, ^45^. It was also noted that some studies used the same control group for comparisons with multiple FGR subgroups^39^, ^43^. Diagnostic criteria for FGR generally centered on EFW < 10^th^ percentile, frequently in conjunction with abnormal Doppler velocimetry or Delphi consensus criteria in more recent publications. In the study of Pacheco Silva *et al*.^34^, FGR was defined as EFW < 10^th^ percentile with normal uterine artery Doppler findings. The authors then divided their cases into two groups: EFW < 3^rd^ percentile and EFW between the 3^rd^ and 10^th^ percentiles. In our analysis, we included as FGR cases only those pregnancies with EFW < 3^rd^ percentile.

The gestational age at MPI assessment varied, allowing for robust subgroup analysis based on the timing of FGR onset (early *vs* late). For the six studies^33^, ^36^, ^37^, ^38^, ^43^, ^45^ in which data were reported as median with IQR, range or 95% CI, conversions to mean ± SD are summarized in Table S3.

### Risk of bias for included studies

**Figure 2:**
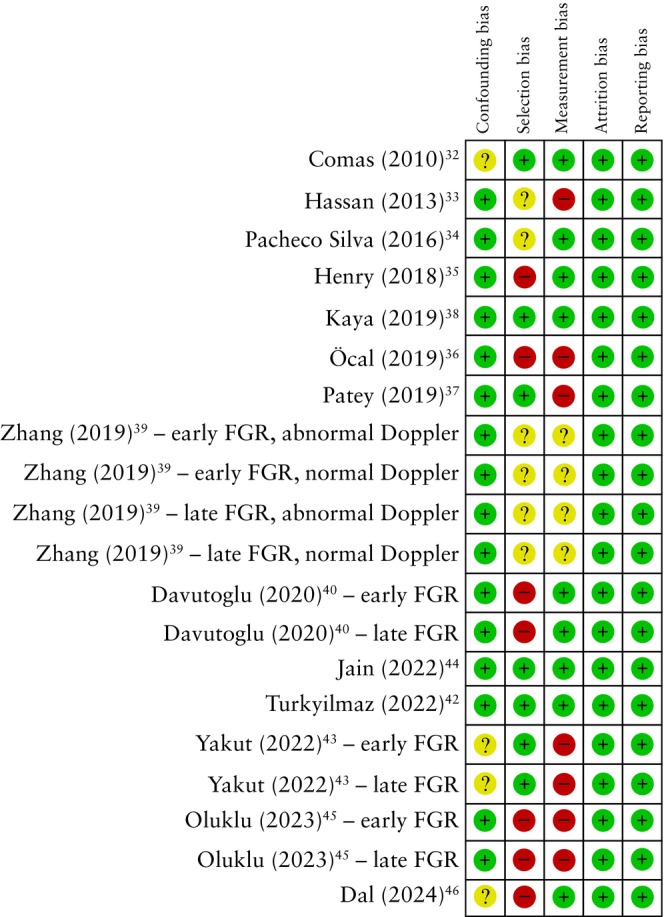
Risk‐of‐bias assessment for included studies. Only first author is given for each study. Green, low risk of bias; yellow, unclear risk of bias; red, high risk of bias. FGR, fetal growth restriction.

The risk of bias for each of the 15 included studies was assessed using criteria adapted from the Cochrane ROBINS‐I tool (Figure 2). Overall, the risk of bias varied across studies and domains. Risk of bias due to confounding was rated as unclear for three studies^32^, ^43^, ^46^, largely due to limited reporting on adjustment for potential confounders. Other studies were rated as low risk in this domain. Risk of bias in the selection of participants was considered high for five studies^35^, ^36^, ^40^, ^45^, ^46^. This was frequently related to broad FGR definitions (e.g. EFW < 10^th^ percentile without mandatory Doppler abnormality) or unclear methodology for selection of controls. Other studies presented unclear or low risk in this domain. Risk of bias in the measurement of FGR status and cardiac parameters was scored as high for studies in which data were reported as medians^33^, ^36^, ^37^, ^43^, ^45^. Two studies also received unclear or high risk ratings in this domain because they presented data for multiple FGR subgroups, which were treated as independent ‘substudies’ in our meta‐analysis, despite sharing baseline features such as enrollment center and treating physicians^39^, ^40^. Risk of attrition bias and reporting bias were considered to be low for all studies.

### Meta‐analysis of fetal myocardial functional parameters

#### Left ventricular function in FGR

The meta‐analysis of left ventricular functional parameters revealed distinct and statistically significant alterations in fetuses affected by FGR compared with their appropriately grown, healthy counterparts.

**Figure 3:**
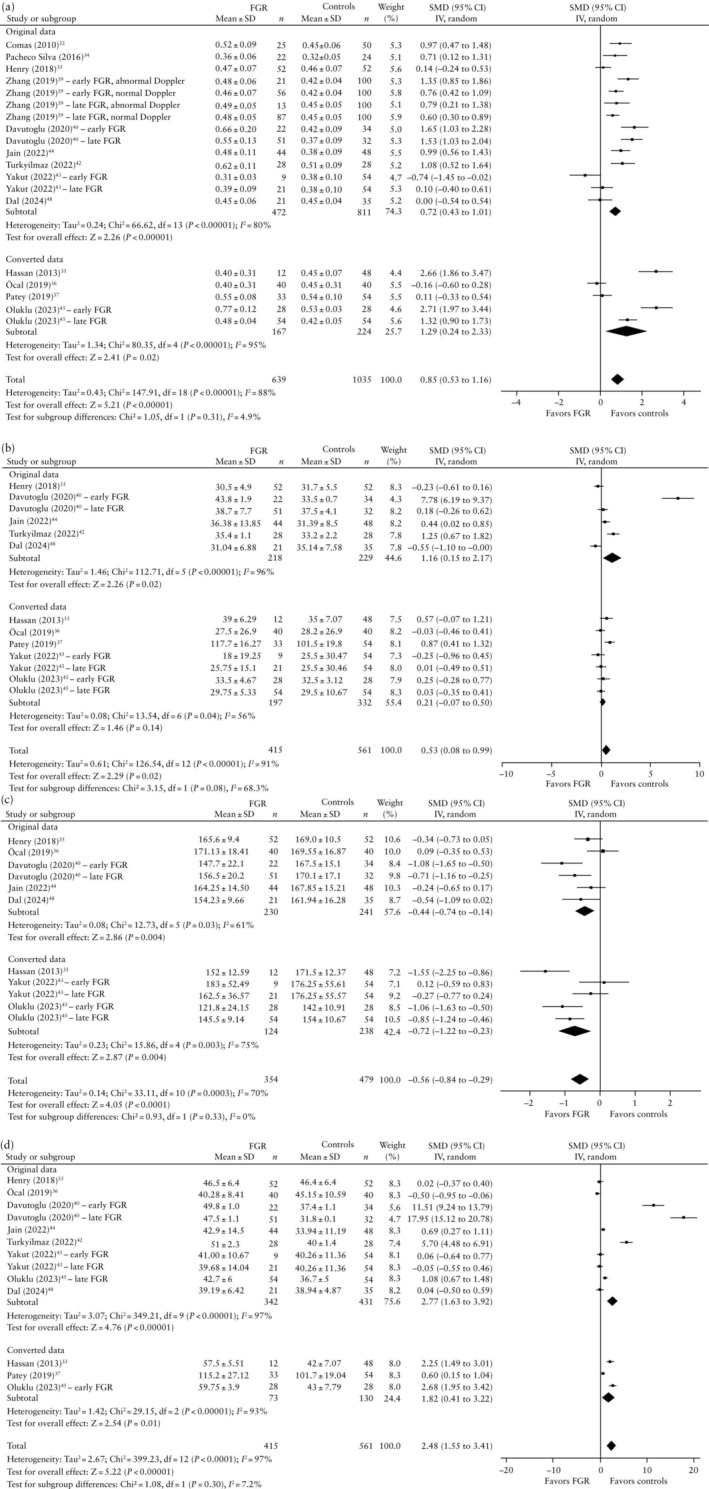
*Continued*. Forest plots summarizing comparison between growth‐restricted (FGR) and control fetuses of left ventricular parameters: (a) myocardial performance index, (b) isovolumetric contraction time, (c) ejection time and (d) isovolumetric relaxation time. Analyses are stratified by format of raw data in original publication: mean ± SD (original data) or median with interquartile range, range or 95% CI (converted data). Only first author is given for each study. IV, inverse variance; SMD, standardized mean difference.

A global assessment of left ventricular performance using the left ventricular myocardial performance index (LVMPI), incorporating data from 19 distinct datasets (13 unique studies), including 639 FGR cases and 1035 controls, demonstrated that LVMPI was significantly higher in the FGR group (SMD, 0.85 (95% CI, 0.53–1.16); *P* < 0.00001) (Figure 3a). This finding is indicative of an overall impairment in left ventricular performance in FGR. However, this result was accompanied by high heterogeneity across the contributing studies (*I* ^2^ = 88%).

To explore this heterogeneity, subgroup analyses were performed. When stratified by the type of data presentation, studies that originally reported mean ± SD values (14 datasets, 472 FGR cases, 811 controls) yielded a pooled SMD of 0.72 (95% CI, 0.43–1.01) (*I* ^2^ = 80%) (Figure 3a). For those studies in which data were converted from other formats (five datasets, 167 FGR cases, 224 controls), the pooled SMD was 1.29 (95% CI, 0.24–2.33) (*I* ^2^ = 95%). The difference between these subgroups was not statistically significant (*P* = 0.31). Analysis by timing of FGR onset also showed consistent findings between subgroups (*P* = 0.14) (Figure S1). Early‐onset FGR (eight datasets, 195 FGR cases, 438 controls) was associated with a significantly higher LVMPI compared with controls (SMD, 1.24 (95% CI, 0.62–1.86); *I* ^2^ = 90%). This pattern was mirrored in late‐onset FGR (eight datasets, 308 FGR cases, 457 controls), in which LVMPI was also significantly elevated compared with controls (SMD, 0.69 (95% CI, 0.30–1.08); *I* ^2^ = 83%).

Examining the individual components of the LVMPI, the left ventricular isovolumetric contraction time (LVICT) was assessed across 13 distinct datasets (10 unique studies), including 415 FGR cases and 561 controls (Figure 3b). The pooled data confirmed that FGR fetuses exhibited a significantly prolonged LVICT (SMD, 0.53 (95% CI, 0.08–0.99); *P* = 0.02; *I* ^2^ = 91%). On subgroup analysis by data presentation type, studies that originally reported mean ± SD values (six datasets, 218 FGR cases, 229 controls) yielded a pooled SMD of 1.16 (95% CI, 0.15–2.17) (*I* ^2^ = 96%), whereas those for which data were converted (seven datasets, 197 FGR cases, 332 controls) showed a more modest SMD of 0.21 (95% CI, −0.07 to 0.50) (*I* ^2^ = 56%); the *P*‐value for this subgroup difference was 0.08. A trend towards greater prolongation of LVICT was observed in early‐onset FGR (four datasets, 83 FGR cases, 164 controls; SMD, 1.92 (95% CI, 0.16–3.69); *I* ^2^ = 96%) compared with late‐onset FGR (six datasets, 208 FGR cases, 257 controls; SMD, 0.29 (95% CI, −0.18 to 0.76); *I* ^2^ = 83%); the *P*‐value for this subgroup difference was 0.08 (Figure S2).

Conversely, the left ventricular ejection time (LVET), derived from 11 distinct datasets (eight unique studies), including 354 FGR cases and 479 controls, was found to be significantly shorter in the FGR cohort (SMD, −0.56 (95% CI, −0.84 to −0.29); *P* < 0.0001; *I* ^2^ = 70%) (Figure 3c). Subgroup analyses by data presentation type (original data *vs* converted data: SMD, −0.44 (95% CI, −0.74 to −0.14) *vs* − 0.72 (95% CI, −1.22 to −0.23); *P* = 0.33) and timing of FGR onset (early *vs* late: SMD, −0.90 (95% CI, −1.53 to −0.27) *vs* − 0.63 (95% CI, −0.88 to −0.38); *P* = 0.42) did not reveal any significant differences (Figures 3c and S3).

Pooled data from 13 distinct datasets (10 unique studies), including 415 FGR cases and 561 controls, demonstrated a marked and highly significant prolongation of left ventricular isovolumetric relaxation time (LVIRT) in FGR fetuses compared with controls (SMD, 2.48 (95% CI, 1.55–3.41); *P* < 0.00001; *I* ^2^ = 97%) (Figure 3d). Subgroup analysis by data presentation type did not reveal a significant difference (*P* = 0.30): studies that originally reported mean ± SD values (10 datasets, 342 FGR cases, 431 controls) yielded a pooled SMD of 2.77 (95% CI, 1.63–3.92) (*I* ^2^ = 97%), whereas studies with converted data (three datasets, 73 FGR cases, 130 controls) yielded a pooled SMD of 1.82 (95% CI, 0.41–3.22) (*I* ^2^ = 93%). Regarding FGR onset, both early‐onset FGR (four datasets, 71 FGR cases, 164 controls; SMD, 3.82 (95% CI, 1.28–6.36); *I* ^2^ = 97%) and late‐onset FGR (six datasets, 208 FGR cases, 257 controls; SMD, 3.37 (95% CI, 1.72–5.02); *I* ^2^ = 98%) exhibited a significant prolongation of LVIRT, with no significant difference between subgroups (*P* = 0.77) (Figure S4).

In contrast, the left E/A ratio, derived from seven datasets, including 241 FGR cases and 280 controls, did not show a significant difference between FGR fetuses and healthy controls (SMD, −0.40 (95% CI, −0.87 to 0.07); *P* = 0.09; *I* ^2^ = 85%) (Figures S5 and S6).

#### Right ventricular function in FGR

Assessment of right ventricular parameters also suggested functional alterations in FGR fetuses, although analyses were based on fewer studies and often showed high heterogeneity.

**Figure 4:**
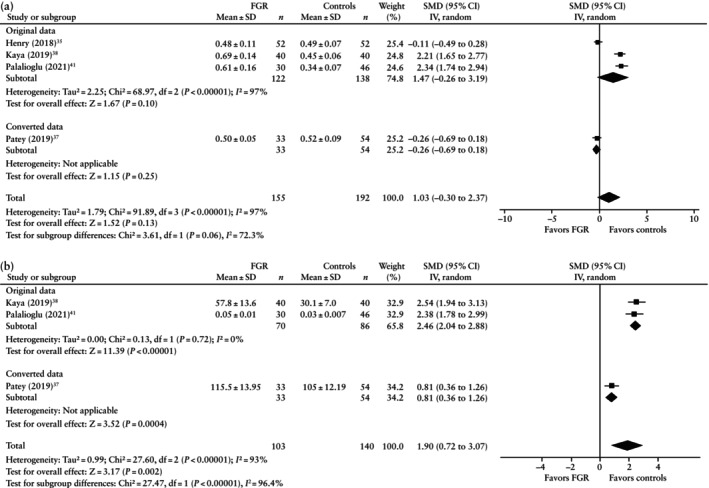
Forest plots summarizing comparison between growth‐restricted (FGR) and control fetuses of right ventricular parameters: (a) myocardial performance index and (b) isovolumetric relaxation time. Analyses are stratified by format of raw data in original publication: mean ± SD (original data) or median with interquartile range, range or 95% CI (converted data). Only first author is given for each study. IV, inverse variance; SMD, standardized mean difference.

The right ventricular myocardial performance index (RVMPI), evaluated from four studies involving 155 FGR cases and 192 controls, showed a pooled SMD of 1.03 (95% CI, −0.30 to 2.37), which did not achieve overall statistical significance (*P* = 0.13; *I* ^2^ = 97%) (Figure 4a). The three studies that originally reported mean ± SD values (122 FGR cases, 138 controls) demonstrated a more robust elevation in RVMPI among FGR cases (SMD, 1.47 (95% CI, −0.26 to 3.19); *I* ^2^ = 97%) compared with the single study with converted data (33 FGR cases, 54 controls; SMD, −0.26 (95% CI, −0.69 to 0.18)), but the difference between subgroups did not reach significance (*P* = 0.06). Stratifying by timing of FGR onset, the single study on early‐onset FGR (30 FGR cases, 46 controls) reported a significant elevation in RVMPI among cases (SMD, 2.34 (95% CI, 1.74–2.94)) (Figure S7). For late‐onset FGR (two studies, 73 FGR cases, 94 controls), the pooled SMD was 0.97 (95% CI, −1.44 to 3.38) (*I* ^2^ = 98%), which was not statistically significant. The difference between subgroups was non‐significant (*P* = 0.28).

Meta‐analysis of three studies (103 FGR cases, 140 controls) showed that right ventricular ICT trended towards prolongation in FGR fetuses (SMD, 0.97 (95% CI, −0.02 to 1.97); *P* = 0.06; *I* ^2^ = 92%) (Figures S8 and S9). The two studies that originally reported mean ± SD values showed a larger effect (70 FGR cases, 86 controls; SMD, 1.42 (95% CI, 0.57–2.27); *I* ^2^ = 82%) compared with the single study with converted data (33 FGR cases, 54 controls; SMD, 0.10 (95% CI, −0.33 to 0.53)); the *P*‐value for this subgroup difference was 0.007.

Data for right ventricular ejection time (RVET) from three studies (122 FGR cases, 138 controls) indicated a trend towards shorter RVET in FGR fetuses (SMD, −1.64 (95% CI, −3.31 to 0.03); *P* = 0.053; *I* ^2^ = 97%) (Figures S10 and S11). No significant difference (*P* = 0.81) was observed between the two studies that originally reported mean ± SD values (82 FGR cases, 98 controls; SMD, −1.54 (95% CI, −4.32 to 1.24); *I* ^2^ = 98%) and the single study with converted data (40 FGR cases, 40 controls; SMD, −1.88 (95% CI, −2.41 to −1.35)).

The right ventricular isovolumetric relaxation time (RVIRT), assessed in three studies (103 FGR cases, 140 controls), was significantly prolonged in FGR fetuses (SMD, 1.90 (95% CI, 0.72–3.07); *P* = 0.002; *I* ^2^ = 93%) (Figures 4b and S12). The two studies (70 FGR cases, 86 controls) that originally reported mean ± SD values yielded an SMD of 2.46 (95% CI, 2.04–2.88), which was larger than the SMD of 0.81 (95% CI, 0.36–1.26) from the single study with converted data (33 FGR cases, 54 controls); the *P‐*value for this subgroup difference was < 0.00001.

Finally, for the right E/A ratio, data from three studies (103 FGR cases, 140 controls) showed a difference between FGR fetuses and controls, although it only just approached significance (SMD, 0.43 (95% CI, −0.002 to 0.86); *P* = 0.051; *I* ^2^ = 63%) (Figures S13 and S14).

### Sensitivity analysis according to FGR diagnostic criteria

To address the potential impact of heterogeneity in FGR definitions, we performed a sensitivity analysis for each primary outcome measure including only the subset of studies that used stricter diagnostic criteria for FGR (AC/EFW < 3^rd^ percentile, or AC/EFW < 10^th^ percentile in conjunction with abnormal Doppler velocimetry or slow fetal growth). Therefore, only 10 studies were included^32^, ^33^, ^34^, ^37^, ^38^, ^39^, ^41^, ^42^, ^43^, ^44^. This analysis confirmed that, compared with controls, LVMPI was significantly higher in the FGR group (SMD, 0.76 (95% CI, 0.43–1.10); *P* < 0.00001), LVICT was significantly higher in the FGR group (SMD, 0.50 (95% CI, 0.09–0.90); *P* = 0.02), LVIRT was significantly higher in the FGR group (SMD, 1.42 (95% CI, 0.39–2.46); *P* = 0.007), RVET was significantly lower in the FGR group (SMD, −2.40 (95% CI, −3.47 to −1.33); *P* < 0.0001) and RVIRT was significantly higher in the FGR group (SMD, 1.90 (95% CI, 0.72–3.07); *P* = 0.002) (Figures S15–S19). No significant differences were found for other parameters.

### Publication bias

Visual inspection of funnel plots was conducted for outcomes with 10 or more datasets, namely LVMPI, LVICT, LVET and LVIRT (Figures S20–S23). The funnel plots for LVMPI (19 datasets) and LVIRT (13 datasets) exhibited some asymmetry. Egger's linear regression test revealed no significant asymmetry for LVICT (13 datasets; intercept, 2.72 (95% CI, −1.63 to 7.07); *P* = 0.21) or LVET (11 datasets; intercept, −2.57 (95% CI, −6.16 to 1.02); *P* = 0.15). However, significant asymmetry was detected for LVMPI (intercept, 2.45 (95% CI, 0.24–4.66); *P* = 0.033) and LVIRT (intercept, 9.39 (95% CI, 3.02–15.76); *P* = 0.006), suggesting potential publication bias or other small‐study effects for these two parameters.

## DISCUSSION

This systematic review and meta‐analysis provides the most comprehensive quantitative evidence to date on biventricular cardiac dysfunction in FGR. By synthesizing data from 15 studies, we demonstrate not only that global myocardial performance is impaired in FGR but, critically, we also identify the prolongation of the IRT as the most robust and profound alteration in both ventricles. This finding positions IRT as a key pathophysiological marker of early diastolic dysfunction and myocardial energetic compromise in FGR, potentially offering greater clinical sensitivity compared with traditional parameters.

Our primary finding is the significant elevation of the LVMPI in FGR fetuses compared with controls (SMD, 0.85), an observation consistent across early‐ and late‐onset FGR phenotypes. This overarching impairment of global left ventricular function aligns with the understanding that the fetal heart undergoes significant adaptive and, ultimately, maladaptive changes in response to the chronic hypoxic and undernourished environment characteristic of FGR^7^, ^8^, ^47^. Investigating the components of the LVMPI provides deeper physiological insights. The LVICT was significantly prolonged in FGR (SMD, 0.53), an effect more pronounced in early‐ *vs* late‐onset FGR (*P* = 0.08), suggesting that, in more severe forms of FGR, the ventricle requires a longer period to overcome the increased systemic vascular resistance. Conversely, the LVET was significantly shorter in FGR fetuses compared with controls (SMD, −0.56), indicating either reduced stroke volume or a compensatory mechanism to maintain cardiac output by increasing heart rate.

The most profound alteration observed was the substantial prolongation of the LVIRT in FGR (SMD, 2.48). Impaired myocardial relaxation is a hallmark of diastolic dysfunction and, in the context of FGR, this is likely a multifactorial consequence of chronic hypoxia and substrate deprivation. Myocardial relaxation is an energy‐intensive process, heavily reliant on adenosine triphosphate (ATP) for calcium reuptake^11^, ^12^. Documented reductions in fetal oxygen and glucose uptake in FGR pregnancies^48^, coupled with evidence of placental mitochondrial dysfunction^49^, strongly suggest a state of myocardial bioenergetic compromise. This energy deficit would directly impair relaxation efficiency, leading to a prolonged IRT. Furthermore, chronic pressure overload and hypoxia can induce myocardial remodeling and increased stiffness, which would further mechanically delay ventricular relaxation^10^, ^50^. The magnitude and consistency of LVIRT prolongation render it a key pathophysiological indicator of cardiac stress, potentially offering a more direct measure of myocardial compromise compared with global MPI or arterial Doppler indices. Notably, the conventional left E/A ratio did not show a significant difference between FGR cases and controls, suggesting that MPI components, particularly IRT, are more sensitive markers of early diastolic changes in FGR. Right ventricular assessment, although based on fewer studies, also pointed towards cardiac dysfunction in FGR. Although the overall pooled RVMPI was not statistically significant, RVIRT was significantly prolonged in FGR fetuses compared with controls (SMD, 1.90), mirroring the left‐sided findings and indicating biventricular compromise, which is biologically plausible given the increased afterload faced by the right ventricle in FGR^37^.

This meta‐analysis has several strengths, such as the evaluation of MPI components for both ventricles, the inclusion of a substantial number of studies and participants (e.g. over 600 FGR cases for LVMPI) and adherence to PRISMA guidelines with preregistration of the protocol. Furthermore, to mitigate the risk of including unreliable data, we performed a strict trustworthiness assessment, which resulted in the exclusion of one study due to concerns regarding an author's retraction record.

However, the inherent heterogeneity observed in most outcomes is a significant constraint, likely reflecting variability in FGR definitions, disease severity and MPI measurement techniques across studies. The necessary conversion of data from median to mean for some studies, while performed using validated methods, may have introduced imprecision, as suggested by subgroup analyses in which the magnitude of the SMD differed between original and converted data. The risk‐of‐bias assessment also identified concerns in some studies regarding participant selection, measurement of exposure/outcome and confounding. Thus, caution should be exercised when interpreting the magnitude of these findings, although the direction of effect remained consistent across meta‐analyses and on sensitivity analysis. Also, the smaller number of studies reporting right ventricular data limits the robustness of our conclusions for right ventricular parameters.

Furthermore, the analysis included studies that employed different Doppler modalities, with some assessing conventional MPI via pulsed‐wave Doppler and others using tissue Doppler imaging. This introduces another layer of methodological variability, as tissue Doppler imaging may offer different sensitivity for detecting subtle myocardial dysfunction. The heterogeneity in ultrasound equipment and specific software settings used across the 15 studies, as highlighted by Lobmaier *et al*. and Peixoto *et al*., also represents a significant, unquantifiable source of variation that could influence MPI measurements and contribute to the high statistical heterogeneity observed in our results^51^, ^52^.

The consistent finding of impaired MPI, driven by marked IRT prolongation, suggests that these echocardiographic parameters, particularly LVIRT, hold considerable promise as adjunctive tools in the clinical surveillance of FGR. A MPI profile showing significant IRT prolongation, especially if worsening over time, could signal failing cardiac adaptation before overt changes in standard arterial Doppler indices or biophysical profile scores become apparent. This ‘early warning’ capability is particularly relevant for late‐onset FGR, in which umbilical artery Doppler may be normal despite underlying fetal hypoxia. Such information could refine risk stratification and contribute to more individualized decisions regarding the optimal timing of delivery. The findings of Jain *et al*.^44^ support this by linking impaired MPI to adverse perinatal outcome. Furthermore, antenatal identification of cardiac dysfunction could facilitate proactive neonatal care and long‐term cardiovascular follow‐up for these at‐risk infants^10^, ^48^.

Future research should focus on large, prospective, multicenter, longitudinal studies with standardized FGR definitions and MPI measurement protocols (ideally Mod‐MPI^15^). A primary goal should be to establish gestational age‐specific prognostic thresholds for MPI and, more specifically, for IRT, correlating these with short‐ and long‐term adverse outcomes, before investigating the added predictive value of MPI over conventional Doppler indices.

In conclusion, this systematic review and meta‐analysis provides comprehensive evidence that FGR is associated with significant alterations in both left and right ventricular myocardial performance. The most consistent, statistically robust and biologically plausible finding is a marked prolongation of the IRT in both ventricles, strongly indicative of early and significant diastolic dysfunction. This impairment is likely a consequence of myocardial energetic compromise and adaptive remodeling due to the chronic adverse intrauterine environment in FGR. Further research is imperative to investigate whether MPI and its components, especially IRT, represent valuable non‐invasive tools that can enhance our understanding and surveillance of cardiac wellbeing in FGR fetuses.

## Supporting information


**Table S1** Trustworthiness assessment of included studies.

**Table S2** Detailed characteristics of 15 studies included in systematic review and meta‐analysis of myocardial function in fetal growth restriction (FGR), including primary outcome data.

**Table S3** Converted mean ± SD values for cardiac parameters from studies reporting data in other formats.

**Figures S1–S4** Forest plots for left ventricular myocardial performance index (Figure S1), left ventricular isovolumetric contraction time (Figure S2), left ventricular ejection time (Figure S3) and left ventricular isovolumetric relaxation time (Figure S4) in growth‐restricted (FGR) *vs* control fetuses, stratified by onset of FGR (early *vs* late).
**Figures S5 and S6** Forest plots for left E/A ratio in growth‐restricted (FGR) *vs* control fetuses, stratified by data presentation type (Figure S5) and onset of FGR (early *vs* late) (Figure S6).
**Figure S7** Forest plot for right ventricular myocardial performance index in growth‐restricted (FGR) *vs* control fetuses, stratified by onset of FGR (early *vs* late).
**Figures S8 and S9** Forest plots for right ventricular isovolumetric contraction time in growth‐restricted (FGR) *vs* control fetuses, stratified by data presentation type (Figure S8) and onset of FGR (early *vs* late) (Figure S9).
**Figures S10 and S11** Forest plots for right ventricular ejection time in growth‐restricted (FGR) *vs* control fetuses, stratified by data presentation type (Figure S10) and onset of FGR (early *vs* late) (Figure S11).
**Figure S12** Forest plot for right ventricular isovolumetric relaxation time in growth‐restricted (FGR) *vs* control fetuses, stratified by onset of FGR (early *vs* late).
**Figures S13 and S14** Forest plots for right E/A ratio in growth‐restricted (FGR) *vs* control fetuses, stratified by data presentation type (Figure S13) and onset of FGR (early *vs* late) (Figure S14).
**Figures S15–S19** Sensitivity analyses for left ventricular myocardial performance index (Figure S15), left ventricular isovolumetric contraction time (Figure S16), left ventricular isovolumetric relaxation time (Figure S17), right ventricular ejection time (Figure S18) and right ventricular isovolumetric relaxation time (Figure S19), including only studies using Delphi consensus definition for diagnosis of fetal growth restriction (FGR).
**Figures S20–S23** Funnel plots for assessment of publication bias for left ventricular myocardial performance index (Figure S20), left ventricular isovolumetric contraction time (Figure S21), left ventricular ejection time (Figure S22) and left ventricular isovolumetric relaxation time (Figure S23). SE, standard error; SMD, standardized mean difference.

## Data Availability

The data that support the findings of this study are available from the corresponding author upon reasonable request.
