# Supplementary material for: Left and right myocardial performance indices in growth‐restricted fetuses: systematic review and meta‐analysis
Source: Ultrasound Obstet Gynecol. 2026 May 11;68(2):174–87. doi: 10.1002/uog.70233 (PMC13432977; doi:10.1002/uog.70233)
Supplement: Supplementary file 1 — Table S1 Trustworthiness assessment of included studies. [file UOG-68-174-s002.docx]

**Table S1**Trustworthiness assessment of included studies.

| **Study** | **Country** | **Study Design** | **Recruitment Period** | **Ethics Approval** | **Sample Size (FGR / Control)** | **Retraction Status** | **Plausibility of Recruitment** | **Trustworthiness Decision** |
| --- | --- | --- | --- | --- | --- | --- | --- | --- |
| **Comas 2010** | Spain | Prospective Case-Control | Not stated | Yes (Local Ethics Committee) | 25/50 | Clear | Yes | **Include** |
| **Hassan 2013** | UK | Prospective Cohort | Dec 2011 – Sept 2012 | Yes (09/H0308/5) | 12/48 | Clear | Yes | **Include** |
| **Nassr 2015** | Egypt | Prospective | Jan 2011 – May 2013 | Yes (Local Ethics Committee) | 71/65 | **Flagged** | Yes | **Exclude** |
| **Pacheco Silva 2016** | Brazil | Prospective Cross-sectional | Jan 2012 – Dec 2013 | Yes (UNIFESP) | 22/24 | Clear | Yes | **Include** |
| **Henry 2018** | Australia | Prospective Cohort | Jun 2012 – Mar 2015 | Yes (8/168, 13/320) | 52/52 | Clear | Yes | **Include** |
| **Öcal 2019** | Turkey | Prospective Case-Control | Dec 2018 – Feb 2019 | Yes (Local Ethics Committee) | 40/40 | Clear | Yes | **Include** |
| **Patey 2019** | UK | Prospective Longitudinal | Feb 2014 – Jun 2016 | Yes (12/LO/0945) | 33/54 | Clear | Yes | **Include** |
| **Kaya 2019** | Turkey | Prospective Cross-sectional | Not stated | Yes (Local Ethics Committee) | 40/40 | Clear | Yes | **Include** |
| **Zhang 2019** | China | Prospective | Oct 2016 – Oct 2017 | Yes (2016-ky-071-01) | 177/200 | Clear | Yes | **Include** |
| **Alici Davutoglu 2020** | Turkey | Prospective Case-Control | Apr 2016 – Sept 2017 | Yes (Local Ethics Committee) | 73/66 | Clear | Yes | **Include** |
| **Palalioglu 2021** | Turkey | Prospective | May 2016 – May 2017 | Yes (2016/2/10) | 30/46 | Clear | Yes | **Include** |
| **Turkyilmaz 2022** | Turkey | Prospective Case-Control | Oct 2020 – Apr 2021 | Yes (Local Ethics Committee) | 28/28 | Clear | Yes | **Include** |
| **Yakut 2022** | Turkey | Prospective Case-Control | Mar 2019 – Sep 2019 | Yes (Local Ethics Committee) | 30/54 | Clear | Yes | **Include** |
| **Jain 2022** | India | Prospective Cohort | Jan 2018 – Jan 2020 | Yes (Local Ethics Committee) | 44/48 | Clear | Yes | **Include** |
| **Oluklu 2023** | Turkey | Prospective Case-Control | Jul 2021 – Mar 2022 | Yes  (E2-21-635) | 82/82 | Clear | Yes | **Include** |
| **Dal 2024** | Turkey | Retrospective | Jun 2023 – Jan 2024 | Yes (2023/871) | 21/35 | Clear | Yes | **Include** |
