# Supplementary material for: Left and right myocardial performance indices in growth‐restricted fetuses: systematic review and meta‐analysis
Source: Ultrasound Obstet Gynecol. 2026 May 11;68(2):174–87. doi: 10.1002/uog.70233 (PMC13432977; doi:10.1002/uog.70233)
Supplement: Supplementary file 2 — Table S2 Detailed characteristics of 15 studies included in systematic review and meta‐analysis of myocardial function in fetal growth restriction (FGR), including primary outcome data. [file UOG-68-174-s004.docx]

**Table S2** Characteristics of 15 studies included in systematic review and meta-analysis of myocardial function in fetal growth restriction (FGR)

| **Study** | **Location** | **Study design** | **Cases (*n*)** | **Controls (*n*)** | **GA at US (weeks)** | **Exclusion criteria** | **FGR diagnostic criteria** | **EFW reference chart** | **Outcome data (cases *vs* controls)** | | | | |
| --- | --- | --- | --- | --- | --- | --- | --- | --- | --- | --- | --- | --- | --- |
|  |  |  |  |  |  |  |  |  | **MPI** | **ICT** | **ET** | **IRT** | **E/A ratio** |
| *LV parameters* |  |  |  |  |  |  |  |  |  |  |  |  |  |
| Comas (2010)^32^ | Spain | Prosp case–control | 25 | 50 | 26–34 | Fetal anomaly*, infection | EFW < p10 + UA-PI > p95 | Figueras^53^ | 0.52 ± 0.09 *vs* 0.45 ± 0.06 | N/A | N/A | N/A | 0.78 ± 0.2 *vs* 0.74 ± 0.1 |
| Hassan (2013)^33^ | UK | Prosp observational | 12 | 48 | 24–32 | Fetal anomaly, aneuploidy, infection | AC < p5 + abnormal UA Doppler | Hadlock^54^ | 0.64 {0.60–0.67} *vs* 0.45 {0.43–0.47} | 39 {35–43} *vs* 34 {33–37} | 152 {144–160} *vs* 171 {168–175} | 57 {54–61} *vs* 42 {40–44} | N/A |
| Pacheco Silva (2016)^34^ | Brazil | Prosp case–control | 22 | 24 | 24–34 | Fetal anomaly* | EFW < p3 | Hadlock^54^ | 0.36 ± 0.06 *vs* 0.32 ± 0.05 | N/A | N/A | N/A | N/A |
| Henry (2018)^35^ | Australia | Cohort/nested case–control | 52 | 52 | 24–38 | Fetal anomaly*, multiple pregnancy, BW ≥ p10 | EFW < p10, or AC < p10 + abnormal UA Doppler | N/A | 0.47 ± 0.07 *vs* 0.46 ± 0.07 | 30.5 ± 4.9 *vs* 31.7 ± 5.5 | 165.6 ± 9.4 *vs* 169.0 ± 10.5 | 46.5 ± 6.4 *vs* 46.4 ± 6.4 | N/A |
| Öcal (2019)^36^ | Turkey | Prosp case–control | 40 | 40 | 29–39 | Maternal systemic disease, fetal anomaly* | EFW < p10 | Hadlock^54^ | 0.4 (0.2–0.6) *vs* 0.4 (0.3–0.7) | 24.5 (13.0–48.0) *vs* 26.0 (13.0–48.0) | 171.13 ± 18.41 *vs* 169.55 ± 16.87 | 40.28 ± 8.41 *vs* 45.15 ± 10.59 | N/A |
| Patey (2019)^37^ | UK | Prosp cohort | 33 | 54 | ≥ 37 | Multiple pregnancy, fetal anomaly*, maternal disease, women in labor | EFW < p10 + Doppler signs of placental dysfunction | N/A | 0.55 (0.50–0.60) *vs* 0.54 (0.48–0.61) | 121 (104–125) *vs* 104 (86–112) | N/A | 118 (95–130) *vs* 101 (90–115) | 0.78 (0.75-0.87) *vs* 0.76 (0.70-0.84) |
| Zhang (2019)^39^ | China | Prosp case–control | 21 | 100 | < 32 | Multiple pregnancy, fetal anomaly, maternal disease | EFW < p10 + abnormal fetal Doppler | Hadlock† | 0.48 ± 0.06 *vs* 0.42 ± 0.04 | N/A | N/A | N/A | N/A |
| Zhang (2019)^39^ | China | Prosp case–control | 56 | 100 | < 32 | Multiple pregnancy, fetal anomaly, maternal disease | EFW < p10 + normal fetal Doppler | Hadlock† | 0.46 ± 0.07 *vs* 0.42 ± 0.04 | N/A | N/A | N/A | N/A |
| Zhang (2019)^39^ | China | Prosp case–control | 13 | 100 | ≥ 32 | Multiple pregnancy, fetal anomaly, maternal disease | EFW < p10 + abnormal fetal Doppler | Hadlock† | 0.49 ± 0.05 *vs* 0.45 ± 0.05 | N/A | N/A | N/A | N/A |
| Zhang (2019)^39^ | China | Prosp case–control | 87 | 100 | ≥ 32 | Multiple pregnancy, fetal anomaly, maternal disease | EFW < p10 + normal fetal Doppler | Hadlock† | 0.48 ± 0.05 *vs* 0.45 ± 0.05 | N/A | N/A | N/A | N/A |
| Davutoglu (2020)^40^ | Turkey | Prosp case–control | 22 | 34 | < 34 | PE, DM, maternal chronic disease, fetal anomaly, multiple pregnancy | EFW < p10 | Hadlock^55^ | 0.66 ± 0.20 *vs* 0.42 ± 0.09 | 43.8 ± 1.9 *vs* 33.5 ± 0.7 | 147.7 ± 22.1 *vs* 167.5 ± 15.1 | 49.8 ± 1.0 *vs* 37.4 ± 1.1 | 0.63 ± 0.14 *vs* 0.74 ± 0.12 |
| Davutoglu (2020)^40^ | Turkey | Prosp case–control | 51 | 32 | ≥ 34 | PE, DM, maternal chronic disease, fetal anomaly, multiple pregnancy | EFW < p10 | Hadlock^55^ | 0.55 ± 0.13 *vs* 0.37 ± 0.09 | 38.7 ± 7.7 *vs* 37.5 ± 4.1 | 156.5 ± 20.2 *vs* 170.1 ± 17.1 | 47.5 ± 1.1 *vs* 31.8 ± 0.1 | 0.75 ± 0.12 *vs* 0.73 ± 0.12 |
| Jain (2022)^44^ | India | Prosp cohort | 44 | 48 | N/A | Multiple pregnancy, fetal anomaly*, abnormal fetal heart rate | AC/EFW < p3 or AC/EFW p3–p10 + abnormal Doppler | N/A | 0.48 ± 0.11 *vs* 0.38 ± 0.09 | 36.38 ± 13.85 *vs* 31.39 ± 8.50 | 164.25 ± 14.50 *vs* 167.85 ± 15.21 | 42.90 ± 14.50 *vs* 33.94 ± 11.19 | N/A |
| Turkyilmaz (2022)^42^ | Turkey | Prosp case–control | 28 | 28 | 32–37 | Hypertensive pregnancy, GDM, fetal anomaly | AC/EFW < p3 or at least two of AC/EFW < p10, AC/EFW crossing centiles by > 2 SD, CPR < p5 or UA-PI > p95 | Hadlock† | 0.62 ± 0.11 *vs* 0.51 ± 0.09 | 35.4 ± 1.1 *vs* 33.2 ± 2.2 | N/A | 51 ± 2.3 *vs* 40 ± 1.4 | 0.79 ± 0.11 *vs* 0.88 ± 0.09 |
| Yakut (2022)^43^ | Turkey | Prosp case–control | 9 | 54 | < 32 | Maternal systemic disease, fetal anomaly*, multiple pregnancy, drug use, PPROM, chorioamnionitis | EFW < p3 or EFW < p10 + UA-PI > p95 or UA-AEDF/UA-REDF/CPR < 1 | N/A | 0.31 ± 0.03 *vs* 0.38 ± 0.10 | 17.0 [8.0–30.0] *vs* 23.0 [8.0–48.0] | 183.0 [153.0–213.0] *vs* 176.0 [140.0–213.0] | 41.00 ± 10.67 *vs* 40.26 ± 11.36 | N/A |
| Yakut (2022)^43^ | Turkey | Prosp case–control | 21 | 54 | ≥ 32 | Maternal systemic disease, fetal anomaly*, multiple pregnancy, drug use, PPROM, chorioamnionitis | EFW < p3 or EFW < p10 + UA-PI > p95 or UA-AEDF/UA-REDF/CPR < 1 | N/A | 0.39 ± 0.09 *vs* 0.38 ± 0.10 | 26.0 [16.0–35.0] *vs* 23.0 [8.0–48.0] | 162.0 [140.0–186.0] *vs* 176.0 [140.0–213.0] | 39.68 ± 14.04 *vs* 40.26 ± 11.36 | N/A |
| Oluklu (2023)^45^ | Turkey | Prosp case–control | 28 | 28 | < 32 | Maternal systemic disease, drug use, nutritional disorder, smoking, umbilical cord or placental anomaly, fetal anomaly* | EFW < p10 | Hadlock^55^ | 0.79 (0.68–0.83) *vs* 0.53 (0.51–0.55) | 33 (31–37) *vs* 33 (30–34) | 118 (110–141) *vs* 142 (135–149) | 60 (57–62) *vs* 42 (39–49) | 0.58 (0.56–0.62) *vs* 0.64 (0.61–0.72) |
| Oluklu (2023)^45^ | Turkey | Prosp case–control | 54 | 54 | ≥ 32 | Maternal systemic disease, drug use, nutritional disorder, smoking, umbilical cord or placental anomaly, fetal anomaly* | EFW < p10 | Hadlock^55^ | 0.48 (0.46–0.51) *vs* 0.41 (0.39–0.46) | 29 (27–34) *vs* 29 (26–33) | 143 (142–154) *vs* 155 (146–160) | 42.7 ± 6 *vs* 36.7 ± 5 | 0.59 (0.54–0.64) *vs* 0.65 (0.62–0.67) |
| Dal (2024)^46^ | Turkey | Retro | 21 | 35 | ≥ 32 | Multiple pregnancy, structural/placental anomaly, GDM, polyhydramnios, chorioamnionitis, PPROM, chronic systemic disease, drug use, aneuploidy, syndromes | AC/EFW < p10 | FMF^56^ | 0.45 ± 0.06 *vs* 0.45 ± 0.04 | 31.04 ± 6.88 *vs* 35.14 ± 7.58 | 154.23 ± 9.66 *vs* 161.94 ± 16.28 | 39.19 ± 6.42 *vs* 38.94 ± 4.87 | N/A |
| *RV parameters* |  |  |  |  |  |  |  |  |  |  |  |  |  |
| Henry (2018)^35^ | Australia | Cohort/nested case–control | 52 | 52 | 24–38 | Fetal anomaly*, multiple pregnancy, BW ≥ p10 | EFW < p10, or AC < p10 + abnormal UA Doppler | N/A | 0.48 ± 0.11 *vs* 0.49 ± 0.07 | N/A | 169.5 ± 9.9 *vs* 170.7 ± 8.1 | N/A | N/A |
| Patey (2019)^37^ | UK | Prosp cohort | 33 | 54 | ≥ 37 | Multiple pregnancy, fetal anomaly*, maternal disease, women in labor | EFW < p10 + Doppler signs of placental dysfunction | N/A | 0.49 (0.48–0.55) *vs* 0.52 (0.47–0.59) | 105 (92–110) *vs* 104 (89–109) | N/A | 119 (103–121) *vs* 106 (96–112) | 0.79 (0.69–0.87) *vs* 0.78 (0.68–0.77) |
| Kaya (2019)^38^ | Turkey | Prosp case–control | 40 | 40 | 34–37 | Maternal systemic disease, fetal anomaly*, multiple pregnancy, arrhythmia, fetal infection, tobacco use | EFW < p3 or EFW < p10 + abnormal Doppler (UA/CPR/UtA-PI) | N/A | 0.69 ± 0.14 *vs* 0.45 ± 0.06 | 51.1 ± 8.1 *vs* 36.9 ± 7.0 | 159.5 (20.8) *vs* 196.7 (29.5) | 57.8 ± 13.6 *vs* 30.1 ± 7.0 | 0.70 (0.12) *vs* 0.59 (0.2) |
| Palalioglu (2021)^41^ | Turkey | Prosp case–control | 30 | 46 | 24–34 | Multiple pregnancy, fetal anomaly*, maternal disease, oligohydramnios, PROM | EFW < p3 or EFW < p10 + abnormal Doppler (UA/CPR/UtA-PI) | N/A | 0.61 ± 0.16 *vs* 0.34 ± 0.07 | 0.05 ± 0.01 *vs* 0.04 ± 0.01 | 0.16 ± 0.01 *vs* 0.19 ± 0.01 | 0.052 ± 0.011 *vs* 0.030 ± 0.007 | 0.74 ± 0.1 *vs* 0.73 ± 0.08 |

Only first author is shown for each study. Data are given as mean ± SD, median {95% CI}, median (interquartile range) or median [range]. *Structural or chromosomal anomaly. †Reference not provided by original study. AC, abdominal circumference; AEDF, absent end-diastolic flow; BW, birth weight; CPR, cerebroplacental ratio; DM, diabetes mellitus; EFW, estimated fetal weight; FMF, Fetal Medicine Foundation; GA, gestational age; GDM, gestational diabetes mellitus; ET, ejection time; ICT, isovolumetric contraction time; IRT, isovolumetric relaxation time; LV, left ventricular; MPI, myocardial performance index; N/A, not available or not reported; p3/p5/p10/p95, 3^rd^/5^th^/10^th^/95^th^ percentile; PE, pre-eclampsia; PI, pulsatility index; PROM, prelabor rupture of membranes; PPROM, preterm prelabor rupture of membranes; Prosp, prospective; REDF, reversed end-diastolic flow; Retro, retrospective; RV, right ventricular; UA, umbilical artery; UtA, uterine artery; US, ultrasound.
