# Supplementary material for: Left and right myocardial performance indices in growth‐restricted fetuses: systematic review and meta‐analysis
Source: Ultrasound Obstet Gynecol. 2026 May 11;68(2):174–87. doi: 10.1002/uog.70233 (PMC13432977; doi:10.1002/uog.70233)
Supplement: Supplementary file 3 — Table S3 Converted mean ± SD values for cardiac parameters from studies reporting data in other formats. [file UOG-68-174-s001.docx]

**Table S3** Converted mean ± SD values for cardiac parameters from studies reporting data in other formats.

| **Study (First Author, Year)** | **Left MPI (Value: Cases vs Controls)** | **Left ICT (Value: Cases vs Controls)** | **Left ET (Value: Cases vs Controls)** | **Left IRT (Value: Cases vs Controls)** | **Left E/A Ratio (Value: Cases vs Controls)** |
| --- | --- | --- | --- | --- | --- |
| Hassan (2013)^33^ | 0.63 ± 0.05 vs 0.45 ± 0.07 | 39 ± 6.29 vs 35 ± 7.07 | 152 ± 12.59 vs 171.5 ± 12.37 | 57.5 ± 5.51 vs 42 ± 7.07 |  |
| Öcal (2019)^36^ | 0.4 ± 0.31 vs 0.45 ± 0.31 | 27.5 ± 26.9 vs 28.2 ± 26.9 |  |  |  |
| Patey (2019)^37^ | 0.55 ± 0.08 vs 0.54 ± 0.10 | 117.7 ± 16.27 vs 101.5 ± 19.8 |  | 115.2 ± 27.12 vs 101.7 ± 19.04 | 0.79 ± 0.09 vs 0.76 ± 0.11 |
| Yakut (2022)^43^ (<32 weeks) |  | 18 ± 19.25 vs 25.5 ± 30.47 | 183.0 ± 52.49 vs 176.25 ± 55.61 |  |  |
| Yakut (2022)^43^ (>32 weeks) |  | 25.75 ± 15.1 vs 25.5 ± 30.46 | 162.5 ± 36.57 vs 176.25 ± 55.57 |  |  |
| Oluklu (2023)^45^ (<32 weeks) | 0.773 ± 0.12 vs 0.53 ± 0.03 | 33.5 ± 4.67 vs 32.5 ± 3.12 | 121.8 ± 24.15 vs 142 ± 10.91 | 59.75 ± 3.9 vs 43 ± 7.79 | 0.585 ± 0.047 vs 0.653 ± 0.086 |
| Oluklu (2023)^45^ (>32 weeks) | 0.48 ± 0.04 vs 0.42 ± 0.05 | 29.75 ± 5.33 vs 29.5 ± 5.33 | 145.5 ± 9.14 vs 154 ± 10.67 |  | 0.59 ± 0.08 vs 0.65 ± 0.04 |
| **Study (First Author, Year)** | **Right MPI (Value: Cases vs Controls)** | **Right ICT (Value: Cases vs Controls)** | **Right ET (Value: Cases vs Controls)** | **Right IRT (Value: Cases vs Controls)** | **Right E/A Ratio (Value: Cases vs Controls)** |
| Patey (2019)^37^ | 0.50 ± 0.05 vs 0.52 ± 0.09 | 103 ± 13.95 vs 101.5 ± 15.23 |  | 115.5 ± 13.95 vs 105 ± 12.19 | 0.78 ± 0.14 vs 0.75 ±0.07 |
| Kaya B (2019)^38^ |  |  | 159.5 ± 16 vs 196.7 ± 22.69 |  | 0.70 ± 0.09 vs 0.59 ± 0.15 |

Values were converted from original data using validated statistical methods (e.g., Wan et al., 2014; Luo et al., 2018) for inclusion in the meta-analysis. ET, ejection time; ICT, isovolumetric contraction time; IQR, interquartile range; IRT, isovolumetric relaxation time; MPI, myocardial performance index; SD, standard deviation.
