# Supplementary material for: Left and right myocardial performance indices in growth‐restricted fetuses: systematic review and meta‐analysis
Source: Ultrasound Obstet Gynecol. 2026 May 11;68(2):174–87. doi: 10.1002/uog.70233 (PMC13432977; doi:10.1002/uog.70233)
Supplement: Supplementary file 4 — Figures S1–S4 Forest plots for left ventricular myocardial performance index (Figure S1), left ventricular isovolumetric contraction time (Figure S2), left ventricular ejection time (Figure S3) and left ventricular isovolumetric relaxation time (Figure S4) in growth‐restricted (FGR) vs control fetuses, stratified by onset of FGR (early vs late). Figures S5 and S6 Forest plots for left E/A ratio in growth‐restricted (FGR) vs control fetuses, stratified by data presentation type (Figure S5) and onset of FGR (early vs late) (Figure S6). Figure S7 Forest plot for right ventricular myocardial performance index in growth‐restricted (FGR) vs control fetuses, stratified by onset of FGR (early vs late). Figures S8 and S9 Forest plots for right ventricular isovolumetric contraction time in growth‐restricted (FGR) vs control fetuses, stratified by data presentation type (Figure S8) and onset of FGR (early vs late) (Figure S9). Figures S10 and S11 Forest plots for right ventricular ejection time in growth‐restricted (FGR) vs control fetuses, stratified by data presentation type (Figure S10) and onset of FGR (early vs late) (Figure S11). Figure S12 Forest plot for right ventricular isovolumetric relaxation time in growth‐restricted (FGR) vs control fetuses, stratified by onset of FGR (early vs late). Figures S13 and S14 Forest plots for right E/A ratio in growth‐restricted (FGR) vs control fetuses, stratified by data presentation type (Figure S13) and onset of FGR (early vs late) (Figure S14). Figures S15–S19 Sensitivity analyses for left ventricular myocardial performance index (Figure S15), left ventricular isovolumetric contraction time (Figure S16), left ventricular isovolumetric relaxation time (Figure S17), right ventricular ejection time (Figure S18) and right ventricular isovolumetric relaxation time (Figure S19), including only studies using Delphi consensus definition for diagnosis of fetal growth restriction (FGR). Figures S20–S23 Funnel plots for assessment of public [file UOG-68-174-s003.docx]

**Figure S1** Forest plot for left ventricular myocardial performance index in growth-restricted (FGR) *vs* control fetuses, stratified by onset of FGR (early *vs* late).


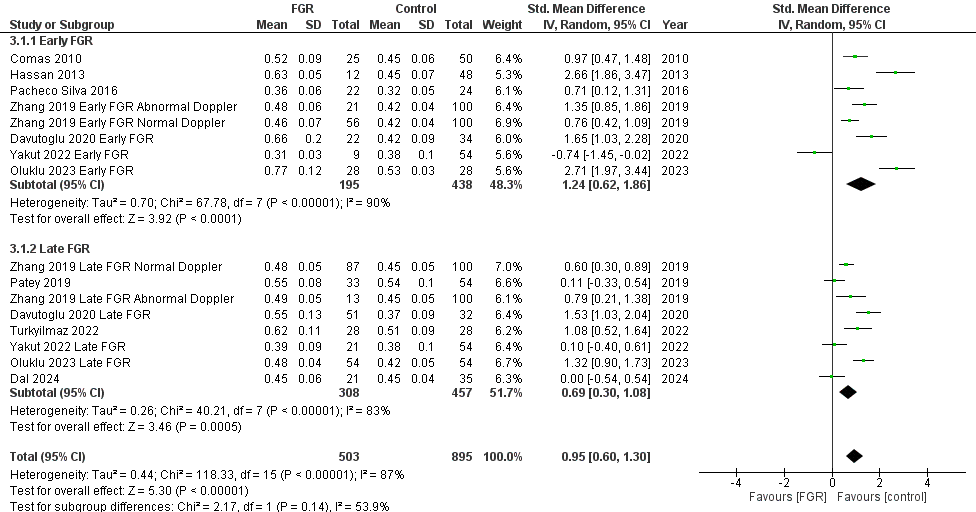


**Figure S2** Forest plot for left ventricular isovolumetric contraction time in growth-restricted (FGR) *vs* control fetuses, stratified by onset of FGR (early *vs* late).


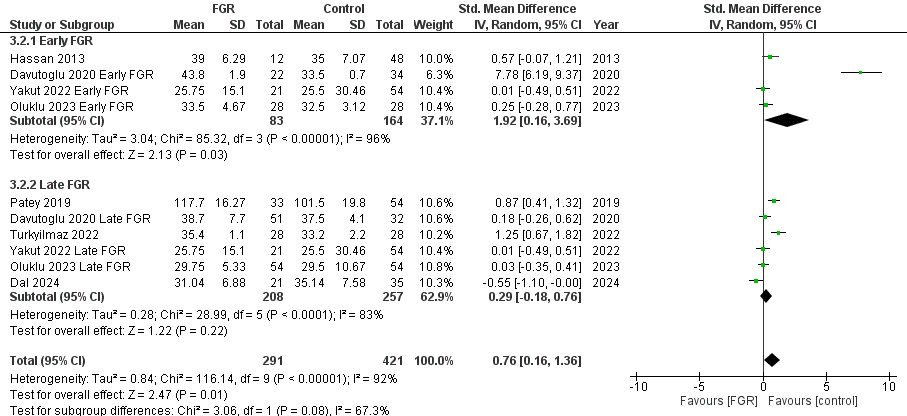


**Figure S3** Forest plot for left ventricular ejection time in growth-restricted (FGR) *vs* control fetuses, stratified by onset of FGR (early *vs* late).


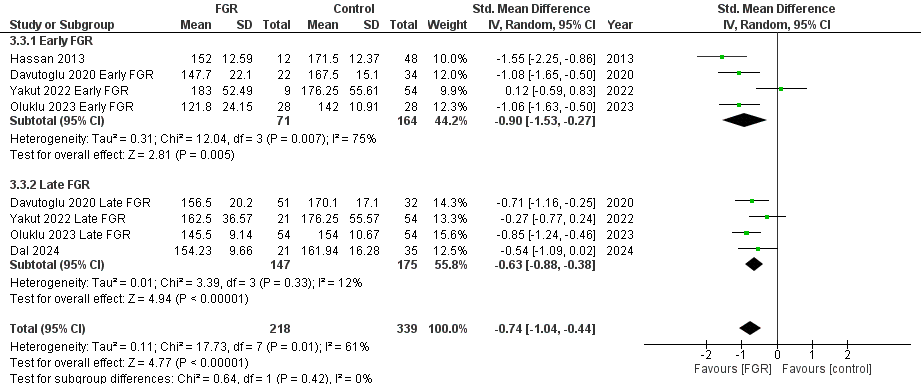


**Figure S4** Forest plot for left ventricular isovolumetric relaxation time in growth-restricted (FGR) *vs* control fetuses, stratified by onset of FGR (early *vs* late).


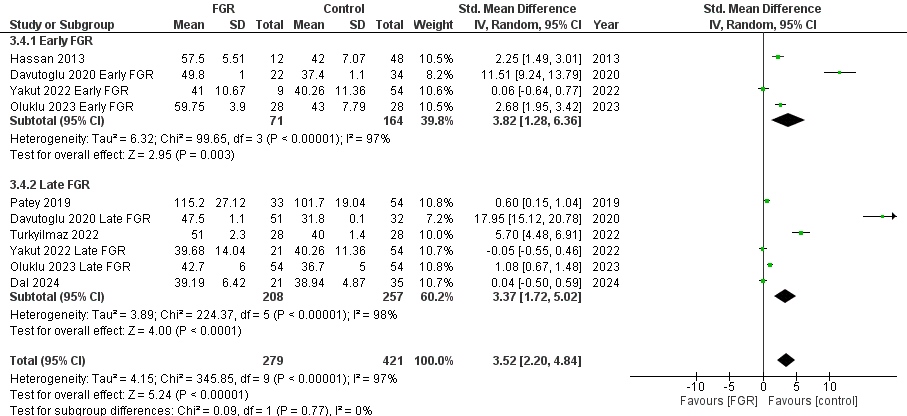


**Figure S5** Forest plot for left E/A ratio in growth-restricted (FGR) *vs* control fetuses, stratified by data presentation type.


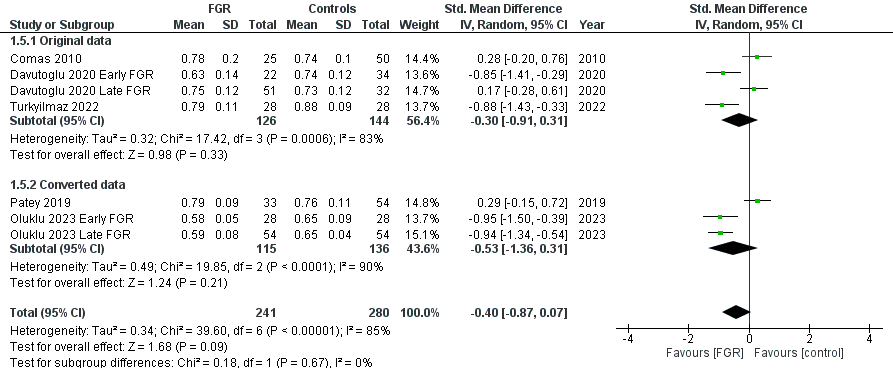


**Figure S6** Forest plot for left E/A ratio in growth-restricted (FGR) *vs* control fetuses, stratified by onset of FGR (early *vs* late).


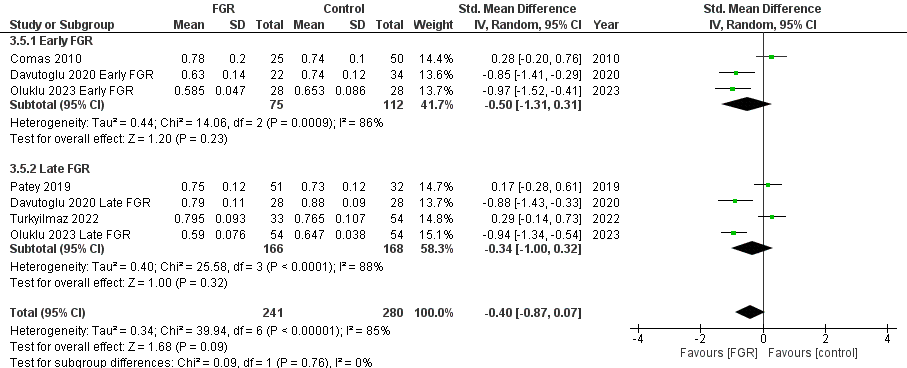


**Figure S7** Forest plot for right ventricular myocardial performance index in growth-restricted (FGR) *vs* control fetuses, stratified by onset of FGR (early *vs* late).


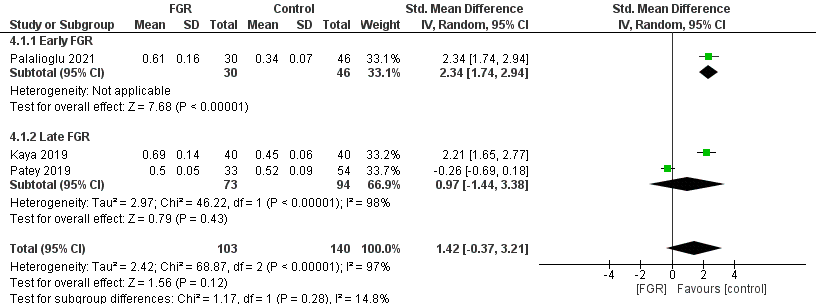


**Figure S8** Forest plot for right ventricular isovolumetric contraction time in growth-restricted (FGR) *vs* control fetuses, stratified by data presentation type.


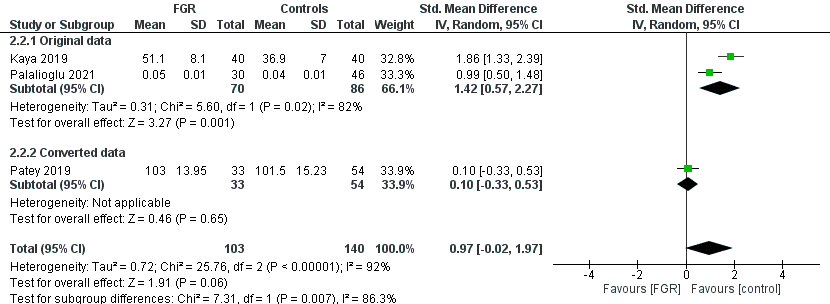


**Figure S9** Forest plot for right ventricular isovolumetric contraction time in growth-restricted (FGR) *vs* control fetuses, stratified by onset of FGR (early *vs* late).


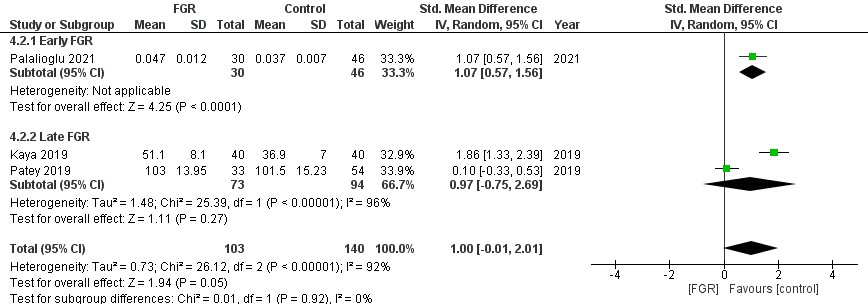


**Figure S10** Forest plot for right ventricular ejection time in growth-restricted (FGR) *vs* control fetuses, stratified by data presentation type.


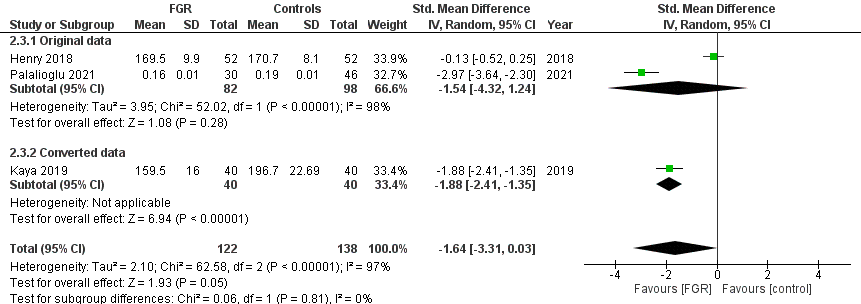


**Figure S11** Forest plot for right ventricular ejection time in growth-restricted (FGR) *vs* control fetuses, stratified by onset of FGR (early *vs* late).


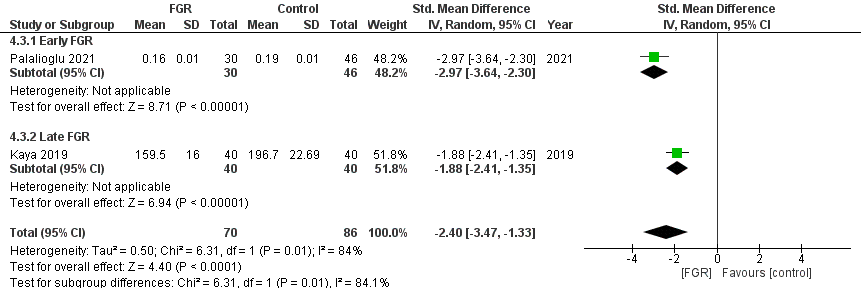


**Figure S12** Forest plot for right ventricular isovolumetric relaxation time in growth-restricted (FGR) *vs* control fetuses, stratified by onset of FGR (early *vs* late).

**
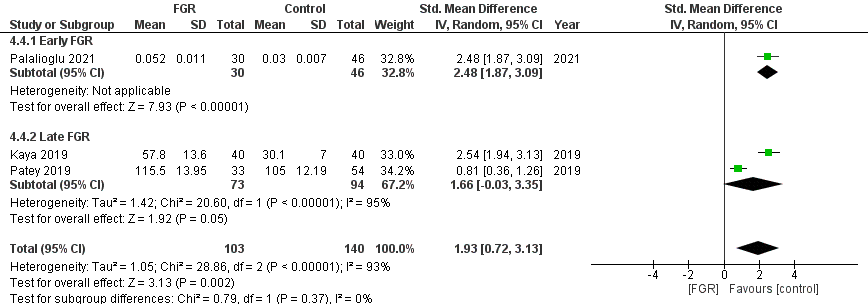
**

**Figure S13** Forest plot for right E/A ratio in growth-restricted (FGR) *vs* control fetuses, stratified by data presentation type.

**
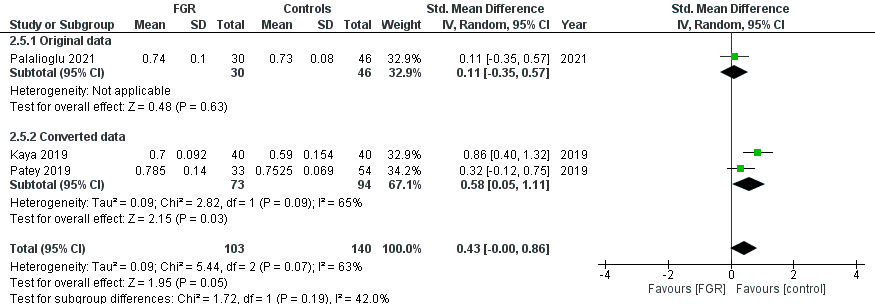
**

**Figure S14** Forest plot for right E/A ratio in growth-restricted (FGR) *vs* control fetuses, stratified by onset of FGR (early *vs* late).

**
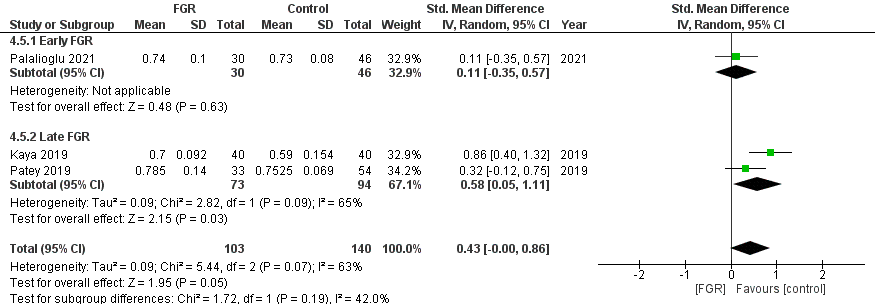
**

**Figure S15** Sensitivity analysis for left ventricular myocardial performance index, including only studies using Delphi consensus definition for diagnosis of fetal growth restriction (FGR).

**
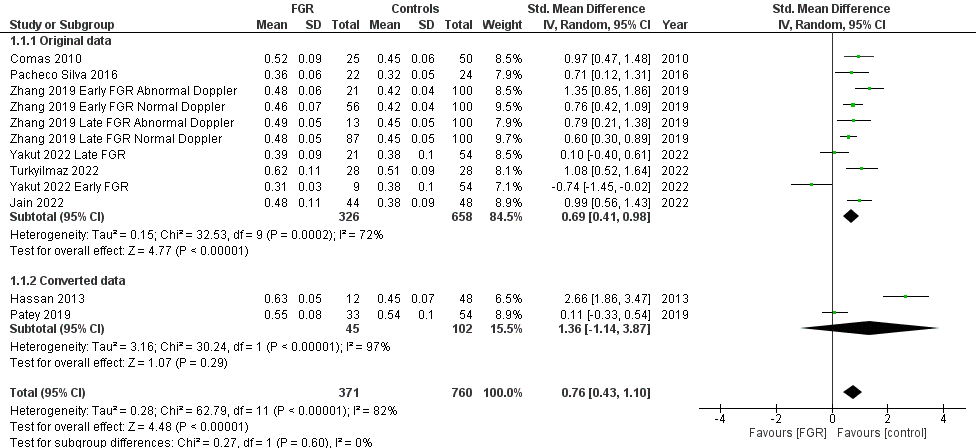
**

**Figure S16** Sensitivity analysis for left ventricular isovolumetric contraction time, including only studies using Delphi consensus definition for diagnosis of fetal growth restriction (FGR).

**
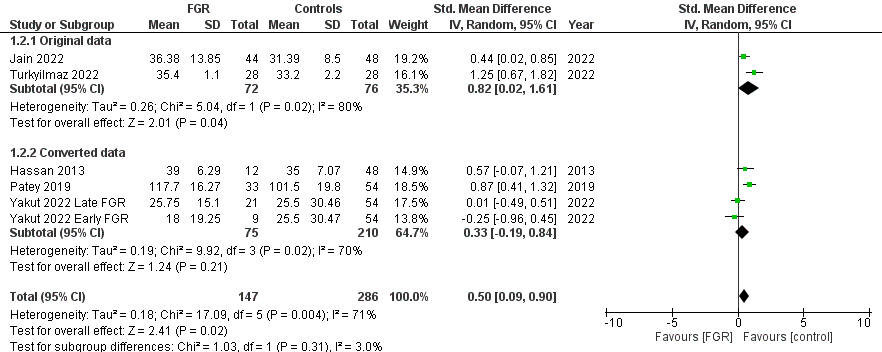
**

**Figure S17** Sensitivity analysis for left ventricular isovolumetric relaxation time, including only studies using Delphi consensus definition for diagnosis of fetal growth restriction (FGR).

**
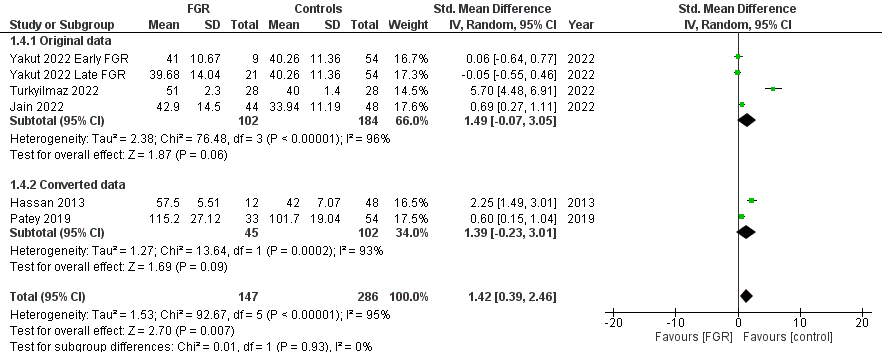
**

**Figure S18** Sensitivity analysis for right ventricular ejection time, including only studies using Delphi consensus definition for diagnosis of fetal growth restriction (FGR).

**
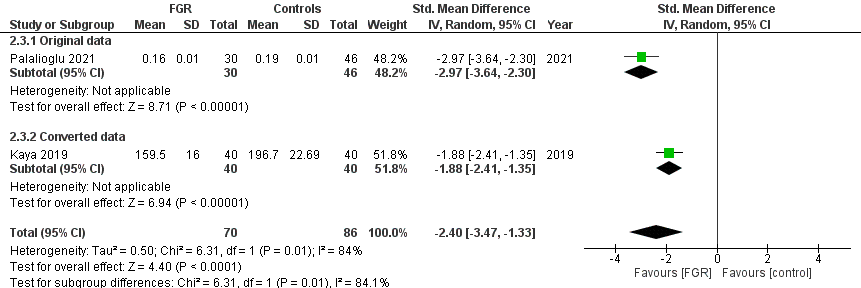
**

**Figure S19** Sensitivity analysis for right ventricular isovolumetric relaxation time, including only studies using Delphi consensus definition for diagnosis of fetal growth restriction (FGR).

**
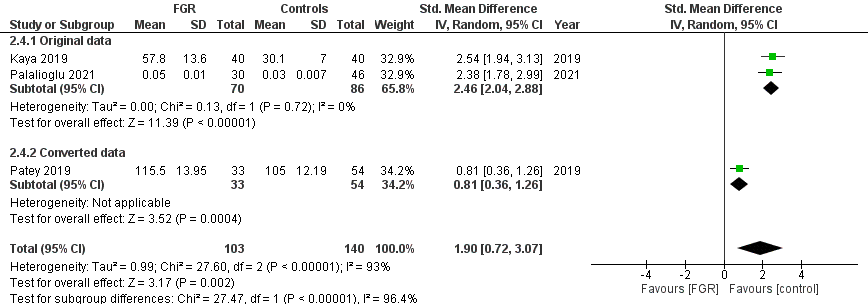
**

**Figure S20** Funnel plot for assessment of publication bias for left ventricular myocardial performance index. SE, standard error; SMD, standardized mean difference.


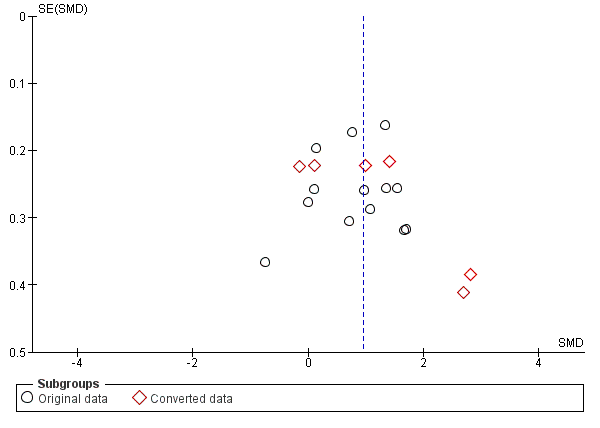


**Figure S21** Funnel plot for assessment of publication bias for left ventricular isovolumetric contraction time. SE, standard error; SMD, standardized mean difference.

**
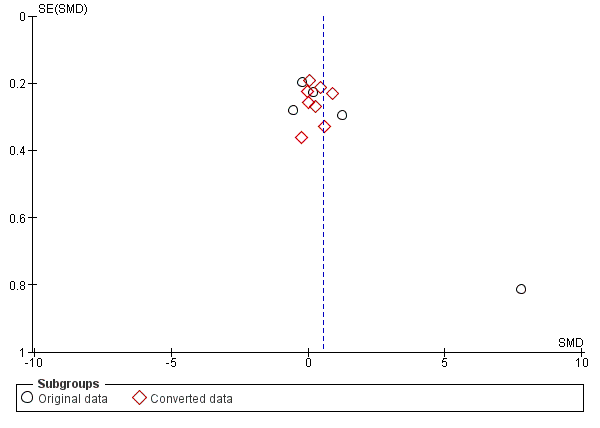
**

**Figure S22** Funnel plot for assessment of publication bias for left ventricular ejection time. SE, standard error; SMD, standardized mean difference.

**
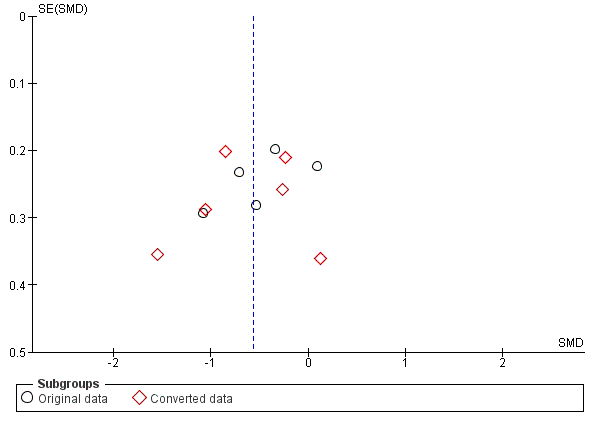
**

**Figure S23** Funnel plot for assessment of publication bias for left ventricular isovolumetric relaxation time. SE, standard error; SMD, standardized mean difference.

**
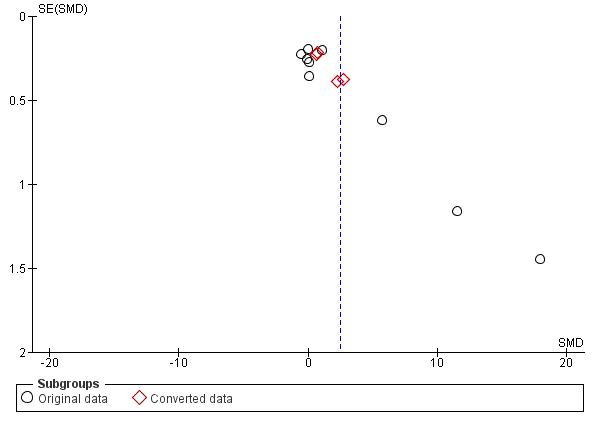
**
